# Carnosol Induces ROS-Mediated Beclin1-Independent Autophagy and Apoptosis in Triple Negative Breast Cancer

**DOI:** 10.1371/journal.pone.0109630

**Published:** 2014-10-09

**Authors:** Yusra Al Dhaheri, Samir Attoub, Gaber Ramadan, Kholoud Arafat, Khuloud Bajbouj, Noushad Karuvantevida, Synan AbuQamar, Ali Eid, Rabah Iratni

**Affiliations:** 1 Department of Biology, College of Science, United Arab Emirates University, Al Ain, United Arab Emirates; 2 Department of Pharmacology & Therapeutics, Faculty of Medicine & Health Sciences, United Arab Emirates University, Al Ain, United Arab Emirates; 3 Department of Biological and Environmental Sciences, College of Arts and Sciences, Qatar University, Doha, Qatar; Toho University School of Medicine, Japan

## Abstract

**Background:**

In this study we investigated the *in vitro* and *in vivo* anticancer effect of carnosol, a naturally occurring polyphenol, in triple negative breast cancer.

**Results:**

We found that carnosol significantly inhibited the viability and colony growth induced G2 arrest in the triple negative MDA-MB-231. Blockade of the cell cycle was associated with increased p21/WAF1 expression and downregulation of p27. Interestingly, carnosol was found to induce beclin1-independent autophagy and apoptosis in MDA-MB-231 cells. The coexistence of both events, autophagy and apoptosis, was confirmed by electron micrography. Induction of autophagy was found to be an early event, detected within 3 h post-treatment, which subsequently led to apoptosis. Carnosol treatment also caused a dose-dependent increase in the levels of phosphorylated extracellular signal-regulated kinase 1 and 2 (pERK1/2). Moreover, we show that carnosol induced DNA damage, reduced the mitochondrial potential and triggered the activation of the intrinsic and extrinsic apoptotic pathway. Furthermore, we found that carnosol induced a dose-dependent generation of reactive oxygen species (ROS) and inhibition of ROS by tiron, a ROS scavenger, blocked the induction of autophagy and apoptosis and attenuated DNA damage. To our knowledge, this is the first report to identify the induction of autophagy by carnosol.

**Conclusion:**

In conclusion our findings provide strong evidence that carnosol may be an alternative therapeutic candidate against the aggressive form of breast cancer and hence deserves more exploration.

## Introduction

Breast cancer continues to be the second leading cause of cancer-related deaths in women. The American Cancer Society estimated nearly 232,670 new cases and about 40 000 deaths estimated due to breast cancer in women for the year 2014 [1].

An approximate of 10 to 15% of breast cancer cases belong to the TNBC (Triple-negative breast cancer) group of cancer. TNBC lack expression of estrogen, progesterone, and the HER-2 epidermal growth factor membrane receptors, are highly aggressive and invasive with poor prognosis of patients and, do not respond to hormonal therapies. Currently, there is no defined standard treatment strategy for prevention of reoccurrence for this disease other than traditional chemotherapy [2].

Apoptosis, major form of programmed cell death, is believed to be a defense mechanism and a tumor suppressor pathway essential for development and maintaining cellular homeostasis. When deregulated apoptosis leads to uncontrolled proliferation of damaged cells and a key role in the pathogenesis and progression of cancer by allowing tumor cells to survive beyond a normal lifespan, but also leads to resistance to chemo or radiotherapy [3]. Apoptosis can be triggered by diverse cellular signals. These include intracellular signals produced in response to cellular stresses, such as increased intracellular Ca^2+^ concentration, DNA damage and high levels of reactive oxygen species (ROS). Extrinsic inducers of apoptosis include bacterial pathogens, toxins, nitric oxide, growth factors, and hormones [4]. Apoptosis is regulated in an orderly way by a series of signaling cascades and occurs by two connected pathways. The extrinsic pathway is initiated by cell surface death receptor stimulation and activation of caspase-8, while the intrinsic pathway involves cytochrome c release from mitochondria and subsequent caspase-9 activation. Activated caspase-8 and-9 activate executioner caspases, including caspase-3, which in turn activate a cytoplasmic endonucleases and proteases that degrade nuclear materials and nuclear and cytoskeletal proteins respectively resulting by eliminating abnormal cells [5]. Evasion from apoptosis is a hallmark of cancer cells which leads to uncontrolled proliferation of damaged cells and contributes to cancer development and enhances resistance to conventional anti-cancer therapies, such as radiation and cytotoxic agents. Most chemotherapeutic agents induce cancer cell death by activation of the apoptotic pathway. However, most of the currently used chemotherapeutics drugs are associated with cytotoxic side-effects and development of chemoresistance [6]–[7].

Although apoptosis is a common mechanism for most of chemotherapeutic drugs that induce cancer cell death, recently, the status of autophagy in cancer therapy has also been given increasing attention. Autophagy is a highly conserved lysosomal degradation pathway by which misfolded or aggregated proteins, damaged organelles and intracellular pathogens are eliminated [8]. Autophagy starts when such unnecessary byproducts and damaged organelles are engulfed into double-membrane vesicles (autophagosomes) and transported to lysosomes where autophagosomes fuse with lysosomes to form single-membrane autolysosomes where the inner engulfed materials are ultimately degraded and recycled. Therefore, autophagy is essential for maintaining homeostasis and seems to play a pro-survival role as well [9]. Apoptosis and autophagy are considered two different events; cross-talk between autophagy and apoptosis exists and the intricate interplay between these two mechanisms is a big challenge for cancer treatment. Autophagy seems to play a role in cancer cell survival and cell death. It contributes to cytoprotective events that help cancer cells to survive and to protect cells from apoptosis [10]. In other circumstances, autophagy can stimulate a pro-death signal pathway in cancer cells. Moreover, under some situations, apoptosis and autophagy can exert synergetic effects, whereas in other conditions autophagy can be triggered only when apoptosis is suppressed [10].

Phytochemicals are natural plant-derived compounds that have been shown to influence in many ways human health. Recently, these natural compounds gained increasing interest for their health promoting properties especially with regard to breast cancer treatment and prevention [11]. Identification and development of new chemotherapeutic agents from plants have gained significant recognition in the field of cancer therapy and become a major area of experimental cancer research. Various phytochemicals present in the diet were found to (i) kill breast cancer cells *in vitro* and (ii) prevent and/or suppress breast cancer progression in various preclinical animal models [12]. Phytochemicals have been shown to target breast cancer development and progression through inhibiting cellular proliferation, suppressing inflammatory processes, arresting cell cycle, inducing apoptosis, modulating gene expression, and inhibiting angiogenesis and invasion potential of many metastatic cancer cell lines. Phytochemical are becoming more attractive for cancer prevention and treatment because they were proven to be very effective, and do not have large side-effect compared to synthetic drugs. Examples of anticancer drugs derived from plants and currently in clinical use include the *vinca* alkaloids vinblastine and vincristine were isolated from *Catharan roseus*, the terpene paclitaxel from *Taxus brevifolia* Nutt, and the DNA topoisomerase I inhibitor camptothecin from *Camptotheca acuminata* [13]. Hence, screening for new natural phytochemicals present a major avenue for the discovery of new and more potent chemopreventive and chemotherapeutic anticancer drugs. Nowadays, much attention is given in understanding the molecular mechanism(s) through which these chemotherapeutic phytochemicals exerts their anti-cancer effects.

Carnosol is a naturally occurring polyphenol (dietary diterpene) found in culinary herbs such as rosemary, sage, and oregano. Studies revealed that carnosol possesses many pharmacological activities, including anti-inflammatory, anti-oxidant, anti-microbial, and anti-cancer properties [14]. Carnosol has been shown to have significant growth inhibitory and cytotoxic effects in several human cancer cell lines and animal models [14]. In fact, carnosol was shown to inhibit the growth of the HT-1080 human fibrosarcoma cells [15], MCF-7 human breast cancer cells [16], PC3 human prostate cancer cells [17] and very recently in human colon cancer cells [18]. Studies reported that carnosol can interfere with multiple signaling pathways that are deregulated in inflammation and cancer [14]. As matter of fact, it has been shown that carnosol inhibited the proliferation, induced G2 arrest and apoptosis in prostate cancer PC3 cells, by activating the intrinsic apoptotic pathway [17]. Moreover, carnosol was found to exert its effect in PC3 cells by affecting several signaling pathways including inhibition of the PI3K/Akt, and activation of the 5'-adenosine monophosphate-activated protein kinase (AMPK) pathway [17]. Carnosol also induced intrinsic mitochondrial apoptotic pathway, through modulating the anti-apoptotic members of the Bcl-2 family of proteins, in acute leukemic cell lines [18].

Additionally, carnosol has been shown to be a potent inhibitor of angiogenesis both *in vitro* and *in vivo* demonstrated by its ability to inhibit differentiation, proliferation, migration and proteolytic potential of endothelial cells [19]. Safety wise, carnosol shown to be safe and well tolerated when administered to animals and most importantly has a selective toxicity toward cancerous cells versus non-tumorigenic cells [16].

In the present study, we investigated the effect of carnosol on the highly proliferative and invasive triple-negative MDA-MB-231 human breast cancer cells. We show that carnosol blocked cell cycle at G2 phase and induced ROS-dependent apoptosis and beciln1-independent autophagy in breast cancer cells. We also show that carnosol inactivated the Signal Transducer and Activator of Transcription 3 (STAT3) signaling pathway and efficiently inhibited tumor growth and metastasis *in vivo* of breast cancer cells.

## Materials and Methods

### Cell culture, chemicals and antibodies

Human breast cancer cells MDA-MB-231 were maintained in Dulbecco Minimal Essential Medium (DMEM) (Hyclone, Cramlington, UK). All media were complemented with 10% fetal bovine serum (FBS) (Hyclone, Cramlington, UK), 100 U/ml penicillin/streptomycin (Hyclone, Cramlington, UK). Carnosol, 2′,7′–dichlorofluorescein diacetate (DCFDA) and tiron were obtained from Sigma Aldrich. Antibodies to p62/SQSTMI were obtained from Abcam (Abcam, Cambridge, UK). Antibodies to LC3, cleaved caspase 3, caspase 8, Bax, Bcl2, p27, and pErk1/2(Th202/Th204), were obtained from Cell Signaling. Antibodies to caspase 9, γH2AX, p21 (WAFA/Cip1), (ser10) histone H3, acetyl histone H3, acetyl histone H4 were obtained from Millipore (Millipore, Hayward, CA, USA). Antibodies to β-actin, pSTAT3 were obtained from Santa Cruz Biotechnology, Inc. Anti-Poly (ADP-ribose) polymerase (PARP) antibody was obtained from BD Pharmingen. Beclin-1 siRNA was purchased from Cell Signaling.

### Measurement of cellular viability

Cells were seeded in triplicate in 12-well plates at a density of 50,000 cells/well. After 24 h of culture, cells were treated with DMSO (vehicle) or various concentrations of carnosol for the indicated time period. Cell viability was measured with the Muse Cell Analyzer (Millipore, Hayward, CA, USA) using the Muse Count and Viability Kit (Millipore, Hayward, CA, USA) which differentially stains viable and dead cells based on their permeability to two DNA binding dyes. Data were presented as proportional viability (%) by comparing the treated group with the untreated cells, the viability of which is assumed to be 100%.

### Cell cycle analysis

The cell cycle distribution analysis in DMSO- or carnosol-treated MDA-MB-231 cells was performed with the Muse Cell Analyzer (Millipore, Hayward, CA, USA) using the Muse cell cycle kit (Millipore, Hayward, CA, USA) according to the manufacturer's instructions. Briefly, cells grown onto 6 cm culture dish were treated with DMSO (vehicle) or various concentrations of carnosol. After 24 h of treatment, cells were collected by trypsinization, washed in PBS and resuspended in complete media and the Muse cell cycle test reagent was then added to each test tube. Cells were then incubated for 30 min at room temperature in the dark. After staining, the cells were processed for cell cycle analysis. Percentage of cells in G0/G1, S and G2/M phases were determined using the FlowJo software.

### Quantification of apoptosis by Annexin V labeling

The apoptosis was examined using the Annexin V & Dead Cell kit (Millipore, Hayward, CA, USA) according to the manufacturer's instructions. Briefly, MDA-MB-231 cells were treated with DMSO (vehicle) or various concentrations of carnosol for 24 h. Detached and adherent cells were collected and incubated with Annexin V and 7-AAD, a dead cell marker, for 20 min at room temperature in dark. The events for live, dead early and late apoptotic cells were counted with the Muse Cell Analyzer (Millipore, Hayward, CA, USA).

### Quantification of multicaspase activity

Multicaspase activity was quantified by using the Muse MultiCaspas*e* assay kit (Millipore, Hayward, CA, USA). MDA-MB-231 cells were seeded at a density of 50,000 cells/well into 12-well plate in triplicate and treated with increasing concentrations of carnosol in presence or absence of Tiron, a ROS scavenger, for 24 h, and multicaspase activity was assessed according to the manufacturer's instructions, using the Muse Cell Analyzer (Millipore, Hayward, CA, USA).

### Determination of intracellular reactive oxygen species (ROS) production

Following treatment with the indicated concentrations of carnosol for the specified time periods, cells were washed with PBS and stained with 5 µM 2′,7′–dichlorofluorescein diacetate (DCFDA) for 30 min at 37°C in the dark. ROS production was determined by analysing fluorescently labelled cells using Nikon Ti U fluorescence microscope.

### Detection of autophagic vacuoles

MDA-MB-231 cells (5×10^4^) were grown in 12 well plates followed by treatment with DMSO (vehicle) or indicated concentrations of carnosol for 24 h. Following treatment cells were washed and stained for autophagic vacuoles using the autophagy detection kit (Abcam, Cambridge, UK) according to the manufacturer's instructions. Fluorescent autophagic vacuoles were examined under Nikon Ti U fluorescence microscope.

### Analysis of the mitochondrial membrane potential

The mitochondrial membrane potential changes were determined with the Muse Cell Analyzer (Millipore) using the Muse MitoPotential kit (Millipore) according to the manufacturer's instructions. Briefly, cells grown in 12-well plates were treated with DMSO (vehicle) or indicated concentrations of Carnosol for 24 h, were collected by trypsinization, washed and incubated with the Muse Mitopotential Dye, a cationic lipophilic dye, for 20 min in a 37°C CO2 incubator. Cells were then incubated with 7-AAD, a dead cell marker, for an additional 5 min at room temperature.

### Transmission electron microscopy

Control and carnosol-treated cells were fixed overnight at 4°C in 2% paraformaldehyde, 2.5% glutaraldehyde in 0.1 M sodium cacodylate buffer pH 7.2 overnight at 4°C, before being post-fixed with 1% OsO_4_ for 1 h. Cells were then dehydrated in a graded ethanol series and embedded in Agar 100 epoxy resin. Ultrathin sections were mounted on Cu grids and stained first with uranyl acetate followed by lead citrate. Sections were observed and photographed under a Philips CM10 Transmission Electron Microscope.

### Colony formation assay in soft agar

Assays were performed in six-well plates as previously described [20]. The lower (base) layer consisted of 1 ml 2.4% Noble agar. The base layer was overlaid with a second layer consisted of 2.9 ml growth medium, 0.3% Noble agar, and 3×10^4^ MDA-MB-231 cells. Growth medium was then added and plates incubated at 37°C. Cells were allowed to grow to form colonies for 14 days before carnosol was added. After 2 weeks, colonies were treated with medium containing 25 µM carnosol for one more week. Following treatment, plates were washed twice with PBS and then colonies were fixed with 10% ice-cold methanol for 10 min and washed once with PBS. Colonies were allowed to stain for 1 h in solution containing 2% Giemsa. The size of the colonies were measured, counted using a microscope (10X) and the colony size was categorized as Large (>200 µM) or small (50–200 µM). Colony sizes were expressed as a percentage of total counted colonies and then compared to the vehicle treated controls (ethanol). The experiment was repeated two times.

### Whole Cell extract and Western Blotting analysis

Cells (1.8×10^6^) were seeded in 100 mm culture dishes and cultured for 24 h before addition of Carnosol at the indicated concentrations. After incubation, cells were washed twice with ice-cold PBS, scraped, pelleted and lysed in RIPA buffer (Pierce) supplemented with protease inhibitor cocktail (Roche) and phosphatase inhibitor (Roche). After incubation for 30 min on ice, cell lysates were centrifuged at 14,000 rpm for 20 min at 4°C. Protein concentration of lysates was determined by BCA protein assay kit (Thermo Scientific) and the lysates were adjusted with lysis buffer. Aliquots of 25 µg of total cell lysate were resolved onto 8–15% SDS-PAGE. Proteins were transferred to nitrocellulose membranes (Thermo Scientific) and blocked for 1 h at room temperature with 5% non-fat dry milk in TBST (TBS and 0.05% Tween 20). Incubation with specific primary antibodies was performed in blocking buffer overnight at 4°C. Horseradish peroxidase-conjugated anti-IgG was used as secondary antibody. Immunoreactive bands were detected by ECL chemiluminescent substrate (Thermo Scientific). Where needed, membranes were stripped in Restore western blot stripping buffer (Thermo Scientific) according to the manufacturer's instructions.

### Knockdown of Beclin-1

MDA-MD-231 cells (250,000) were seeded in 6 cm cell culture dish in serum-containing growth media and allowed to grow. After 24 hr, cells were transfected with SignalSilence Beclin-1 siRNA I (100 nM) using DharmaFECT4 transfection reagent as described by the manufacturer (Thermo Scientific). Cells were transfected for 72 hr at 37°C in 5% CO2 before treating cells with and without 50 µM carnosol in fresh complete media.

### Statistical analysis

Results were expressed as means ± S.E.M. of the number of experiments. A Student's *t*-test for paired or unpaired values was performed and a *p* value of <0.05 was considered statistically significant.

## Results

### Carnosol inhibits the viability of the MDA-MB-231 breast cancer cells

**Figure 1:**
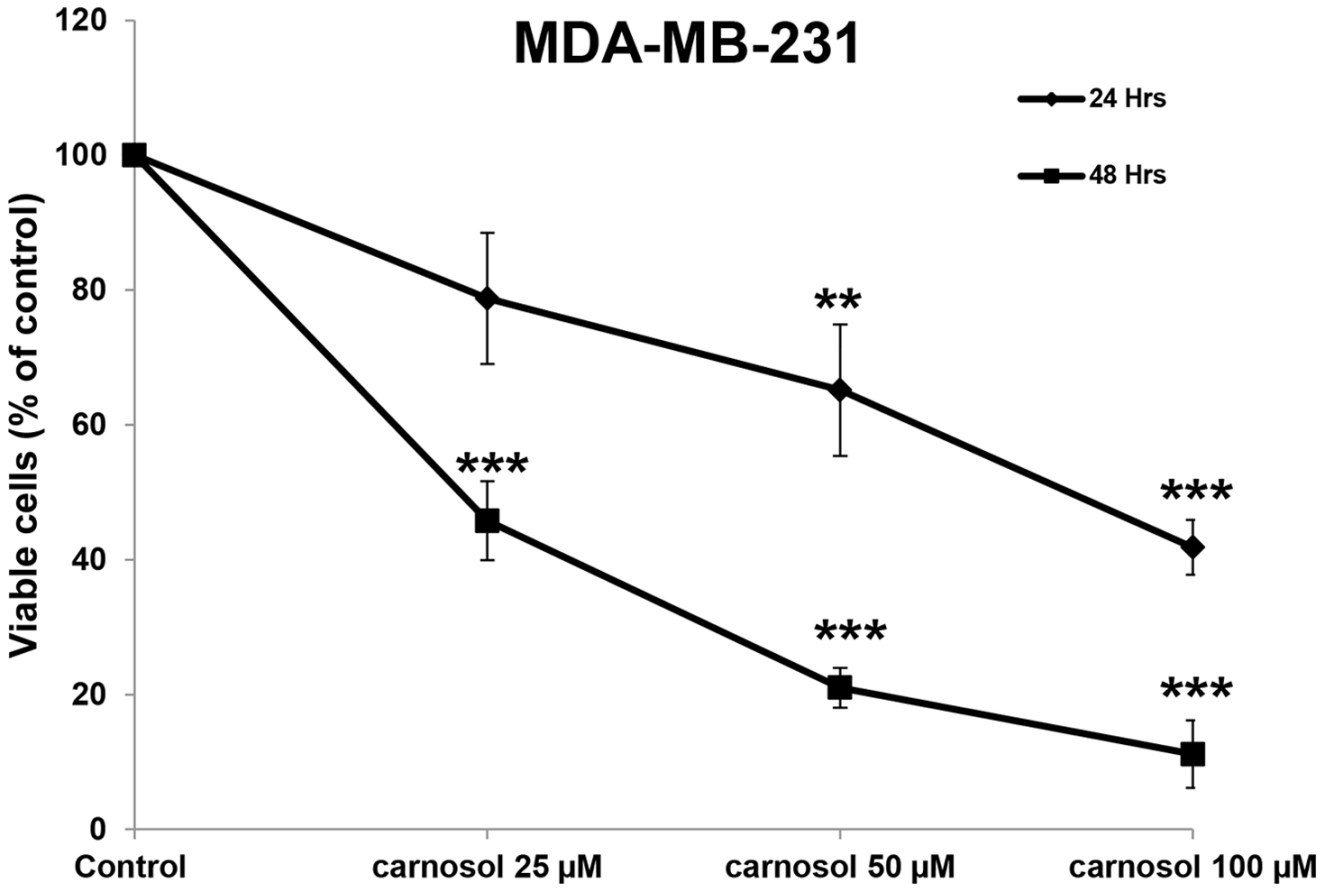
Inhibition of cell viability by carnosol. Exponentially growing MDA-MB-231 were treated with vehicle (DMSO) and the indicated concentrations of carnosol for 24 h and 48 h. Viable cells were assayed with the Muse Cell Analyzer as described by the manufacturer. Data represent the mean ±SEM of at least 3 independent experiments. Student's t test was performed to determine the significance (*p<0.05, **p<0.01 and ***p<0.005).

To examine the anticancer activity of carnosol on breast cancer cells, we first measured the effect of increasing concentrations of carnosol (0, 25, 50 and 100 µM) on the proliferation of the MDA-MB-231 breast cancer cell line (Figure 1). Our results show that the exposure of the MDA-MB-231 to carnosol decreased cellular viability in a concentration- and a time-dependent manner. The IC_50_ (producing half-maximal inhibition) was approximately 83 µM at 24 h and 25 µM at 48 h of treatment.

### Carnosol induces G2-phase cell cycle block in MDA-MB-231 cells

**Figure 2:**
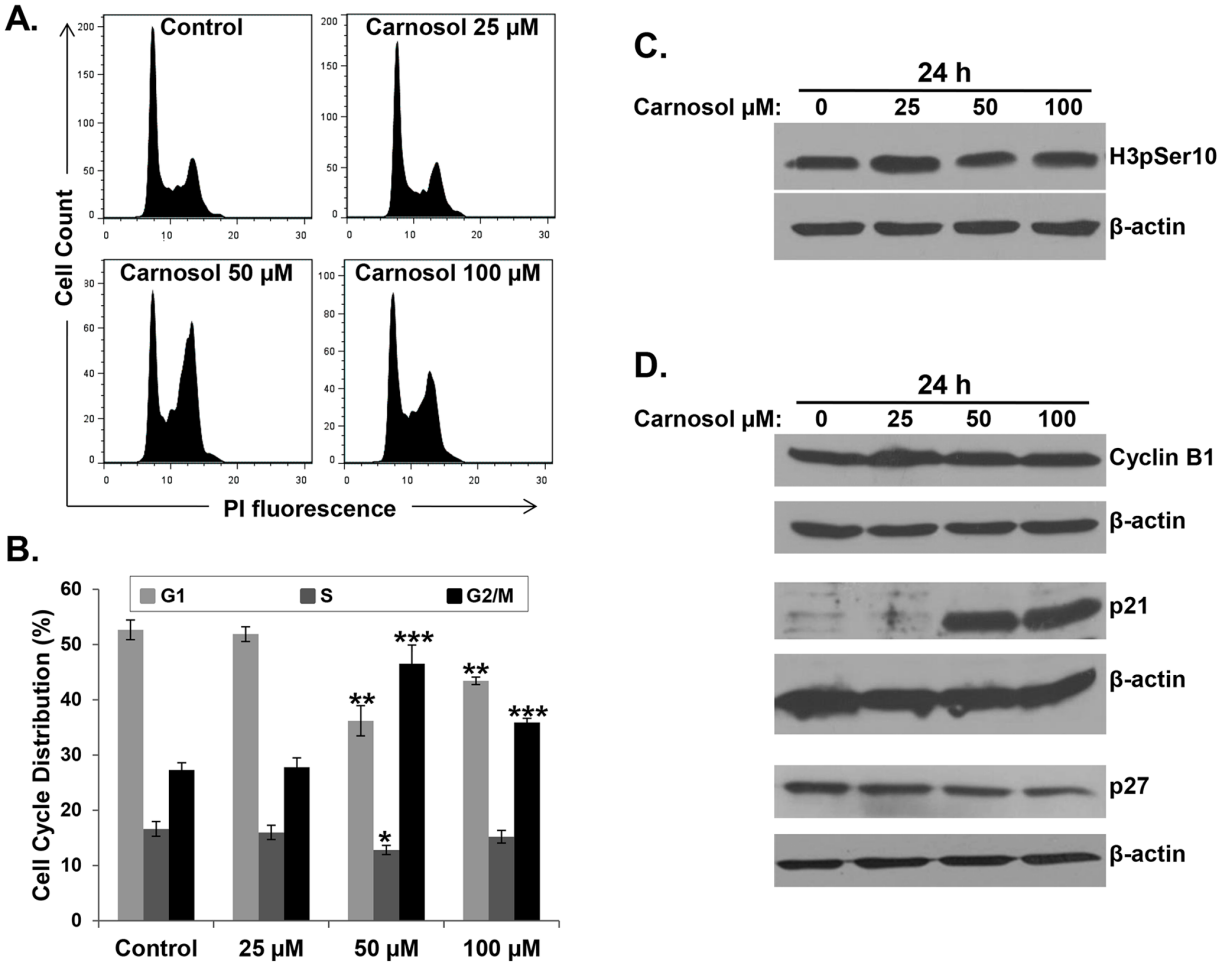
Carnosol induces G2 arrest in MDA-MB-231 cells. (**A–B**) Cell cycle distribution analysis of carnosol-induced G2 cell-cycle arrest. MDA-MB 231 cells were treated with carnosol at the indicated concentrations for 24 h, then analyzed with Muse Cell Analyzer using the Muse cell cycle kit as described in Materials and Methods. (**C–D**) Effect of Carnosol on cell cycle markers. Cells were treated with DMSO or indicated concentrations of carnosol and the expression level of histone H3-p(ser10), Cyclin B1, p21 and p27 was examined by western blotting after 24 post-treatment.

The ability of an anticancer drug to affect cell cycle distribution can provide information regarding its cytotoxic mechanism(s) of action. Moreover, carnosol has been shown to induce G2 block in human prostate cancer PC3 cells [17]. For this reason, we investigated the effect of carnosol on cell cycle distribution in breast cancer cells. MDA-MB 231 cells were treated with indicated concentrations of carnosol for 24 h and subjected to cell cycle analysis. A concentration of 50 and 100 µM of carnosol caused an obvious G/2M arrest on these cells (figure 2A and 2B). Indeed, the population of G2/M increased significantly from 27 in control to 47% and 36% in cell treated with 50 and 100 µM carnosol, respectively, indicating that carnosol-treated MDA-MB-231 cells were arrested in G2/M phase. No obvious change in the cell cycle progression was observed at concentration of 25 µM carnosol after 24 h treatment.

To determine whether carnosol induced cell cycle arrest specifically at mitosis or G2 phase, we examined the phosphorylation status of histone H3 (Ser 10). Histone H3 is phosphorylated at serine 10 during mitosis by aurora kinase and the phosphorylation status of H3 is considered a marker of mitosis [21]. Therefore, we investigated the expression of p(ser10)H3 and found that carnosol treatment did not affect the phosphorylation level of histone H3 in MDA-MB231 cells (Figure 2C). We have also examined the expression of cyclin B1, in carnosol-treated cells. Accumulation of cyclin B1 is well known to play an important role in G2/M transition ant its upregulation invokes mitotic arrest. We found that the level of cyclin B1 remained unchanged in response to carnosol treatment (Figure 2D). Altogether these data confirm a G2 arrest by carnosol in MDA-MB-231 breast cancer cells.

Next, we examined the expression level of the CDK inhibitor, p21 in response to carnosol by Western blotting. The results of a typical experiment shown in figure 2D, demonstrates a significant and dramatic increase of p21 protein only when MDA-MB-231 cells were treated with concentrations (50 and 100 µM) causing cell cycle arrest. No change in p21 protein level was observed with 25 µM carnosol, a concentration that did not affect cell cycle progression. The level of another CDK inhibitor, p27 was also determined in carnosol-treated cells. As it is shown in figure 2D, the level of p27 showed a dose-dependent decrease in MDA-MB-231 cells. Intriguingly, the decrease, although modest, in p27 level was also observed at concentration of 25 µM carnosol. Taken together, our results showed that carnosol-induced cell cycle arrest at G2 phase correlates with p21 overexpression.

### Carnosol induces apoptosis through the intrinsic and extrinsic apoptotic pathways

**Figure 3:**
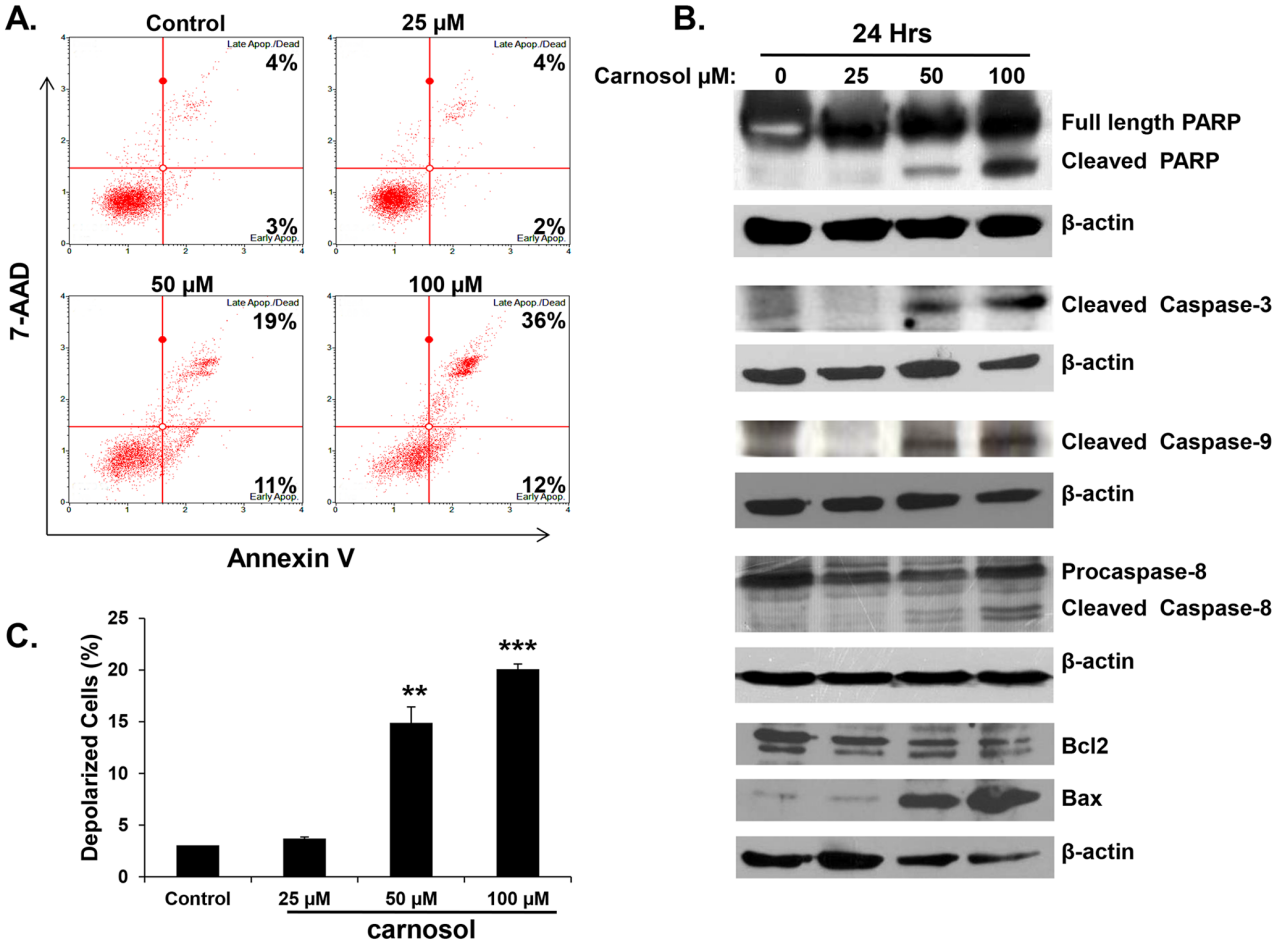
Induction of caspases-mediated apoptosis by carnosol in the MDA-MB-231 cells. (**A**) Carnosol induced apoptosis in the MDA-MB-231 cells. Annexin V binding was carried out using Annexin V & Dead Cell kit (Millipore). Cells were treated with DMSO or various concentration of carnosol for 24 h. Detached and adherent cells were collected and stained with Annexin V and 7-AAD and then the events for early and late apoptotic cells were counted with the Muse Cell Analyzer as described in Materials and Methods. (**B**) Western blot analysis of caspase 3, -9, and -8 activation, PARP cleavage and Bcl1 and Bax expression in MDA-MB-231 cells were treated with increasing concentrations of carnosol (25, 50 and 100 µM) for 24 h. (**C**) Carnosol induces the depolarization of mitochondrial membrane. Mitochondrial membrane potential (MMP) was assessed with the Muse Cell Analyzer using the Muse MitoPotential kit as described in Materials and Methods. Data represent the mean ±SEM of at least 3 independent experiments. Student's t test was performed to determine the significance (*p<0.05, **p<0.01 and ***p<0.005).

Next, we investigated whether the observed growth inhibition upon carnosol treatment was associated with induction of apoptosis. Following 24 hours of treatment, annexin V staining was detected in cells treated with 50 and 100 µM of carnosol (Figure 3A), indicating that these cells are undergoing apoptosis. No necrotic cells were detected at both concentrations. No Annexin V stain was detected at lower concentration (25 µM), indicating absence of apoptosis which is in agreement with the cell viability data. In order to determine the apoptotic pathway induced by carnosol in MDA-MB-231 cells, activation of caspase 3, 8 and 9 along with PARP cleavage was assessed by Western blotting. As it is shown in figure 3B, cleaved PARP and cleaved caspase 3, 8 and 9 increased in concentration-dependent (50 and 100 µM) manner in carnosol-treated cells. Again, neither caspase activation nor PARP cleavage was detected in cells treated with 25 µM carnosol for 24 h. It appears then, that carnosol induces cell death in MDA-MB-231 cells through the activation of the intrinsic and extrinsic pathways of apoptosis by the activation of both caspase 8 and 9.

### Carnosol modulates the expression of Bcl2/Bax proteins and induces mitochondrial membrane potential depolarization in MDA-MB-231 cells

Bcl2 family of proteins which includes Bcl2, and bcl-2 related members such as Bax, plays a crucial role in regulation apoptosis. Therefore, we decided to test the effect of carnosol on the expression level of the anti-apoptotic Bcl2, and pro-apoptotic Bax, proteins. As it is shown in figure 3B (lower panel), carnosol reduced, in dose dependent manner, the level of Bcl2 while increased the level of Bax in MDA-MB-231 cells leading to an increase in the Bax/Bcl2 ratio which is in favor for apoptosis induction.

Mitochondria are known to play a central role to elicit apoptosis in response to many stresses and the loss of mitochondrial membrane potential represents a hallmark for an early event in apoptosis induction. In order to assess for the function of the mitochondria and investigate whether mitochondria are involved in carnosol-induced apoptosis, a change of the mitochondrial membrane potential was analyzed as described in Material and Methods. Results in figure 3C showed a concentration-dependent increase in the number of cells with depolarized mitochondrial membranes, thus suggesting that carnosol causes depolarization of mitochondrial potential which could be linked with the activation of apoptosis in MDA-MB-231 cells.

### Carnosol induces Beclin1-independent autophagy in MDA-MB-231 breast cancer cells

**Figure 4:**
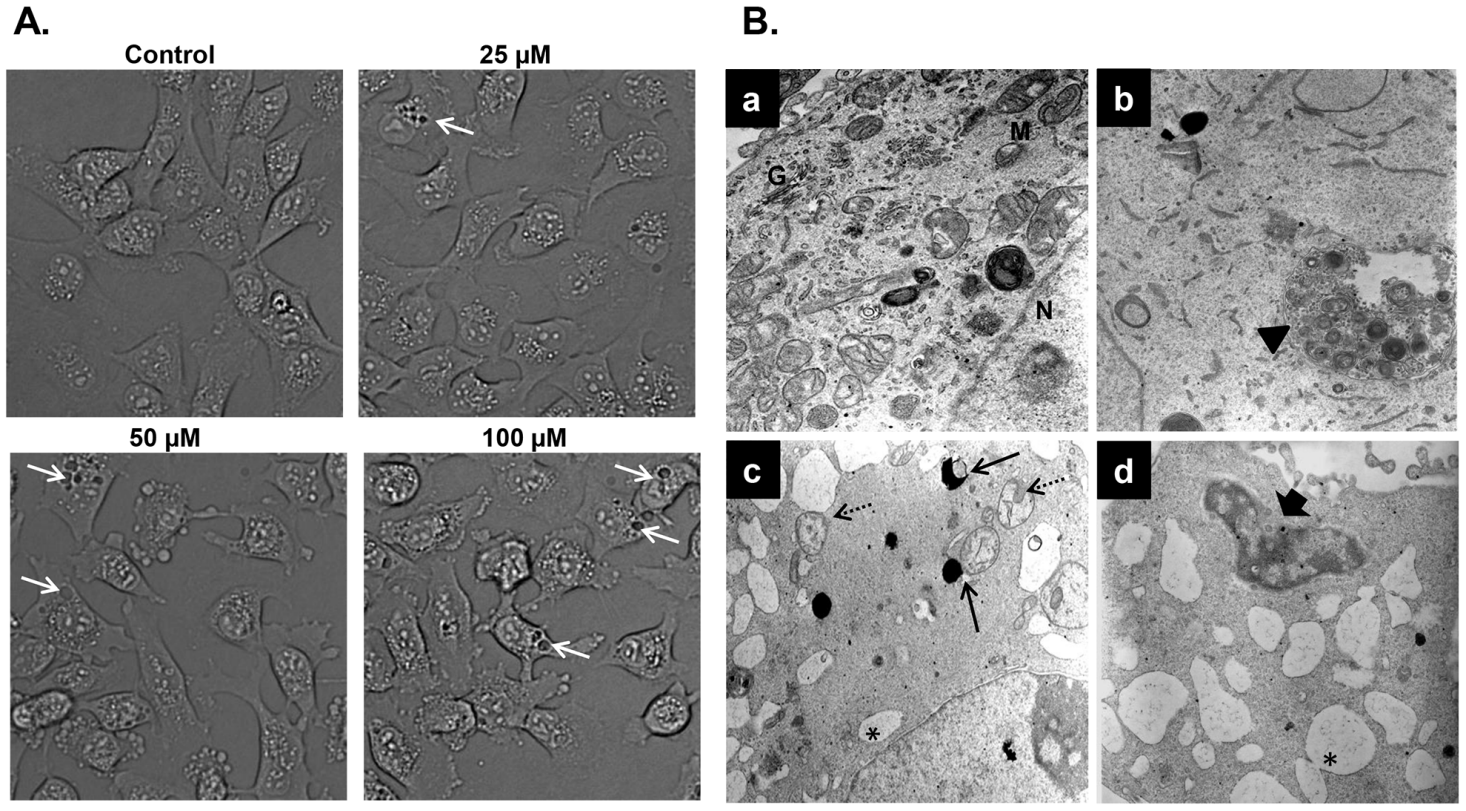
Light and electron microscopy analysis of carnosol-treated MDA-MB-231 cells. (**A**) Micrograph of MDA-MB-231 cells, after 6 h incubation with various concentrations of carnosol. (**B**) Representative electron micrographs of untreated MDA-MB-231 cells (a) and MDA-MB-231 cells treated with 25 (b), 50 (c) and 100 (d) µM carnosol for 24 h.

Light microscopy observation of MDA-MB-231 cells treated with carnosol revealed cytoplasmic vacuolation (Figure 4A) that might indicate that carnosol-induced cell death may be associated with autophagy. In order to determine whether carnosol induces autophagy, we examined by transmission electron microscopy, the ultrastructure of MDA-MB-231 cells treated for 24 h with and without increasing concentrations (25, 50 and 100 µM) of carnosol. As it appears in figure 4B (panel a), control MDA-MB-231 cells showed an intact nuclei (N) and well developed organelles such as endoplasmic reticulum, Golgi and numerous mitochondria. In cells treated with low concentration of carnosol 25 µM, a clearly visible double-membrane surrounded autophagosomes of different sizes engulfing many cellular organelles, including mitochondria can be seen (panel b, arrowhead). Swollen mitochondria were observed as well (data not shown). Very little cytoplasmic vacuoles were found and no obvious sign of cell death was observed at this concentration. At higher concentration of carnosol (50 and 100 µM) the presence of swollen and damaged mitochondria exhibiting disorderly cristae structure (dashed arrow), and very little intact mitochondria could be seen in all cellular sections analyzed. Autophagolysosomes resulting from the fusion of mitochondria and lysosomes were also observed (plain arrow). Such fusion results in the formation of an increasing number of empty vacuoles which then fuses (asterix) to form very large number of macro-vacuoles that occupy most of the cytoplasmic space. These observations suggest that carnosol mediate its effect, at least in part, through induction of mitophagy. Interestingly, very little organelles were present probably due to massive degradation through autophagolysosome. Interestingly, peripheral chromatin condensation and damaged nuclear envelope (large arrow), two characteristics of apoptosis, were also obvious in cells undergoing autophagy which indicates that the two events, apoptosis and autophagy, co-exist in carnosol-treated breast cancer cells.

**Figure 5:**
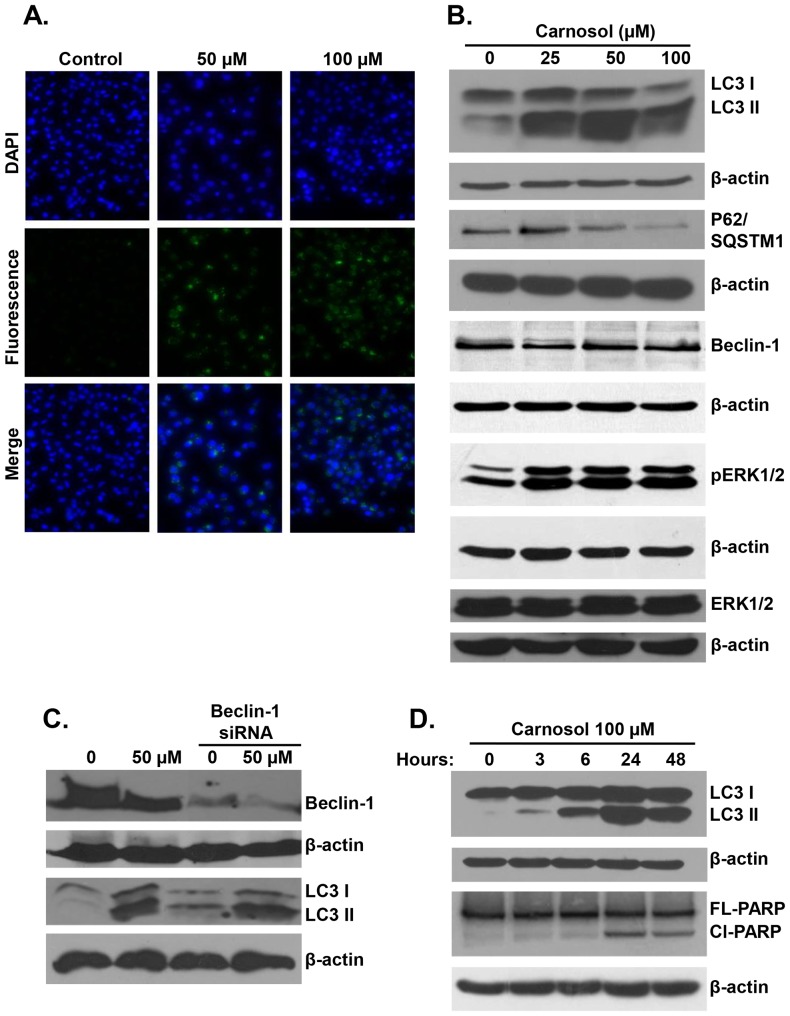
Carnosol induces Autophagy in MDA-B231 cells. (**A**) Carnosol induces the formation of autophagic vacuoles MDA-MB 231 cells observed after 24 h post treatment. MDA-MB-231 cells were seeded at a density of 5×10^4^ cells per well into 12-well plate followed by treatment with DMSO (vehicle) or 50 and 100 µM carnosol. Following treatment cells were washed and stained for autophagic vacuoles using the autophagy detection kit according to manufacturer's instructions. Fluorescent autophagic vacuoles were examined under Nikon Ti U fluorescence microscope. (**B**) Western blotting analysis of LC3II, p62(SQSTM1), Beclin-1 and pERK1/2 in carnosol-treated MD-MB 231 cells. Cells were treated with DMSO or increasing concentration of carnosol for 24 h, then whole cell proteins were extracted and subjected to Western blot analysis, as described in Materials and Methods, for LC3II, 62(SQSTM1), Beclin1, pERK1/2 and β-actin (loading control) proteins. (**C**) carnosol induced autophagy is independent of Beclin1. MDA-MB-231 cells were transiently transfected with siRNA against Beclin1 for 72 hours followed by exposure to carnosol (50 µM) for 24 hours. Whole cell lysates were then probed for LC3II. (**D**) Time-course analysis, by Western blotting, of PARP cleavage and LC3II accumulation in carnosol-treated MDA-MB-231 cells. Cells were treated with 100 µM carnosol and proteins were extracted at the indicated time-point (3, 6, 24 and 48 h) as described in Materials and Methods.

To further confirm the formation of autophagic vacuoles in carnosol-treated cells, we have used a fluorescence marker of autophagy vacuoles as described in Material and Methods. As it is shown, in figure 5A, exposure of MDA-MB-231 cells for 24 h to carnosol led to a concentration-dependent accumulation of autophagic vacuoles, thus further confirming the electron microscopy data. Autophagy is also characterized by the conversion of LC3I (cytosolic form) into a lipidized LC3II (autophagosome membrane-bound form). In order to further confirm autophagy in carnosol-treated MDA-MB-231cells, LC3II accumulation was analyzed by Western blotting in MDA-MB-231 treated with 25, 50 and 100 µM carnosol. As it is shown in figure 5B, carnosol induced a concentration-dependent accumulation of the LC3-II. Interestingly, autophagy is also detected in cells treated with 25 µM carnosol, a concentration that does not induce apoptosis. The expression of another widely used autophagy-specific marker p62(SQSTM1), a ubiquitin-binding protein involved in autophagy and whose level decreases when autophagy flux increases, was also evaluated. As shown in figure 5B, while the concentration of 25 µM show a slight increase in p62 (SQSTM1) expression, a higher concentration of carnosol (50 and 100 µM) leading to massive autophagy, showed a concentration-dependent decrease in p62(SQSTM1). Although, it is widely known from the literature that p62 gets degraded during the process of autophagy and that an increase in p62 is related to cells becoming resistant to autophagy. However, there are also reports showing that an increase in p62 expression is required for autophagy induction [22], [41]–[43]. Hence, the conversion of LC3I/II along with the modulation of p62 (SQSTM1) level confirm the formation of autophagosome in carnosol-treated MDA-MB-231 cells. Next we assessed the expression of Beclin1, an important autophagy effector that plays a key role in autophagosome formation. Western blotting data showed that the level of Beclin1 remained unchanged in response to carnosol, thus suggesting that carnosol induces autophagy that is Beclin1-independent (Figure 5B). To confirm that Beclin-1 is not required for carnosol-induced autophagy, we knocked out beclin-1 protein in MDA-MB-231 cells using Beclin-1-specific siRNA. As it is shown in figure 5C, we found that knock down of Beclin-1 did not inhibit LC3II accucmulation and hence autophagy induced by carnosol. Taken together, these data provide strong evidence that exposure of MDA-MB-231 cells to carnosol triggers Beclin-1 independent autophagy.

Recently, Wong and collaborators showed that ROS-dependent ERK activation is for both apoptosis and beclin1-independent autophagy in many cancer cell lines including the breast cancer MDA-MB-231 cells [22]. This prompted us to measure, by western blotting, the level of activation of ERK1/2 in carnosol-treated MDA-MB-231 cells. Figure 5B, shows a clear dose-dependent increase in pERK1/2 suggesting a possible involvement of ERK activation in carnosol-mediated apoptosis and/or autophagy.

### Autophagy precedes apoptosis in carnosol-treated MDA-MB-231 cells

A lot of studies reported that apoptosis and autophagy can be both activated by anticancer drugs [23], [24]. Our data showed that both apoptosis and autophagy increased in concentration-dependent manner in MDA-MB-231 cells in response to carnosol treatment. In fact we found that while both apoptosis and autophagy were found to coexist in MDA-MB-231 cells treated with high concentration of carnosol (50 and 100 µM); a lower and non-cytotoxic concentration (25 µM) however, showed only sign of autophagy but no caspase activation nor PARP cleavage was detected. Cell death occurred, at this concentration, only after 48 h post-treatment. Based on these observations, it appears then, that upon exposure to carnosol MDA-MB-231, cells first undergoes autophagy and later, due to longer exposure and/or excessive cellular damage, cells progress to apoptosis. In order to confirm this, we have monitored the induction of autophagy and apoptosis over time by performing time-course analysis of these two events. MDA-MB-231 cells were treated for 24 h with 100 µM and apoptosis was detected through PARP cleavage which is downstream of caspase activation, and autophagy through the conversion of conversion of LC3I into LC3II. Figure 5D showed that autophagy was triggered, although modestly, starting 3 h but become more evident 6 h post-treatment. On the other hand, PARP cleavage (Cl-PARP) occurred only 24 h post-treatment. Our data clearly indicates that autophagy preceded apoptosis in carnosol-treated MDA-MB-231 breast cancer cells.

### Carnosol triggers ROS production and induces activation of γH2AX, a marker of double strand breaks, in MDA-MB 231 cells

**Figure 6:**
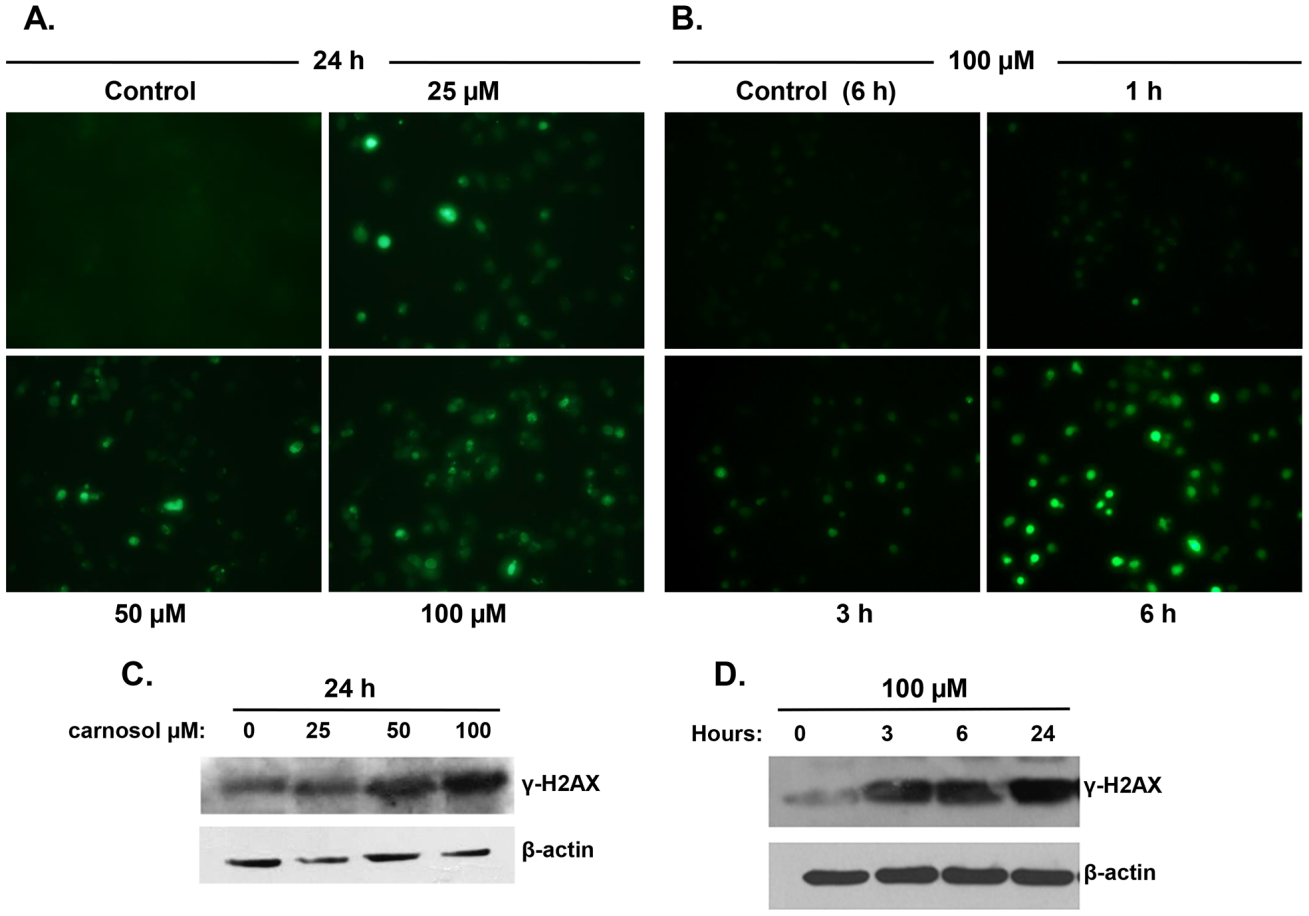
ROS generation and DNA damage accumulation in carnosol-treated MDA-MB-231 cells. (**A**) Carnosol induces ROS generation in MDA-MB-231 cells. Cells were treated with indicated concentrations of carnosol for 24 h and ROS production was analyzed, as described in Material and Methods, using DCFDA fluorescence stain. (**B**) time-course measurement of ROS generation in carnosol-treated MDA-MB-231 cells. Cells were treated with 100 µM carnosol and ROS generation was examined at different time-point (1, 3 and 6 h). (**C–D**) Concentration and time-dependent accumulation of γH2AX, marker of DNA damage, in carnosol-treated cells. DNA damage was evaluated by measuring, by Western blotting, the level γH2AX accumulation using anti-phospho-H2AX (ser 139) antibody.

There are increasing evidences pointing out the involvement of reactive oxygen species (ROS) in the induction of autophagy as well as apoptosis [25], [26]. Therefore we decided to examine the effect of carnosol on ROS production in MDA-MB-231 cells. Toward this, control and treated cells with increasing concentrations of carnosol for 24 h were incubated with 2′,7′–dichlorofluorescein diacetate (DCFDA) and the ROS production was observed under fluorescence microscopy. As it is shown in figure 6A, microscopic observation of carnosol-treated cells showed a significant dose-dependent increase in the intensity of DCF-mediated fluorescence compared with control cells. In order to assess whether ROS production is an early event that leads to autophagy and/or apoptosis, a time-course measurement of ROS production in MDA-MB-231 cells treated with 100 µM carnosol was carried out. Figure 6B shows a significant accumulation of ROS in time-dependent manner that was detected at 3 h post-treatment, which also coincides with the time of LC3II accumulation (Figure 5D). Still, a modest ROS production could be detected as early as 1 h post-treatment. Our data suggests that ROS production might be an early event that possibly triggers or at least contribute in the induction of both autophagy and apoptosis.

Several anticancer drugs were shown to induce apoptosis through DNA damage. Genotoxic DNA damaging agents may activate both membrane death receptors and the endogenous mitochondrial damage pathway leading to cell death via apoptosis [27]. Also, increasing evidences argue in favor of a role of DNA damage in the induction of autophagy; however, the mechanism through which DNA damage triggers autophagy and the role that autophagy plays in the DNA damage response and cellular fate remain unclear. Therefore, we sought, in the first instance, to investigate whether carnosol induced DNA damage in MDA-MB-231 cells. For this purpose, MDA-MB 231 cells were cultured for 24 h in absence or presence of 25, 50 or 100 µM carnosol and DNA damage was determined by measuring the levels of phosphorylated H2AX (γH2AX). Western blotting analysis revealed a concentration-dependent increase in the levels of γH2AX in response to carnosol treatment (Figure 6C), indicating an accumulation of double strand breaks in these cells. A time-course determination of DNA damage induced by 100 µM carnosol revealed that the activation of γH2AX occurred as early as 3 h post-treatment (Figure 6D), a time in which no cell death (data not shown) nor apoptosis were observed, thus ruling out the possibility that the induction of DNA damage is a consequence of DNA fragmentation resulting from caspase activation. From these results, it appears that the induction of apoptosis and/or autophagy correlates with the amount of DNA damage detected, which is dependent on the concentration of carnosol used in the treatment. Autophagy was detected when low amount of DNA damage occurred in the cell, while apoptosis was detected in cells showing excessive DNA damage. These data suggests that DNA damage could account, at least in part, in the induction of both apoptosis and autophagy in carnosol-treated breast cancer cells.

### The ROS scavenger tiron attenuates DNA damage, autophagy and apoptosis in carnosol-treated cells

**Figure 7:**
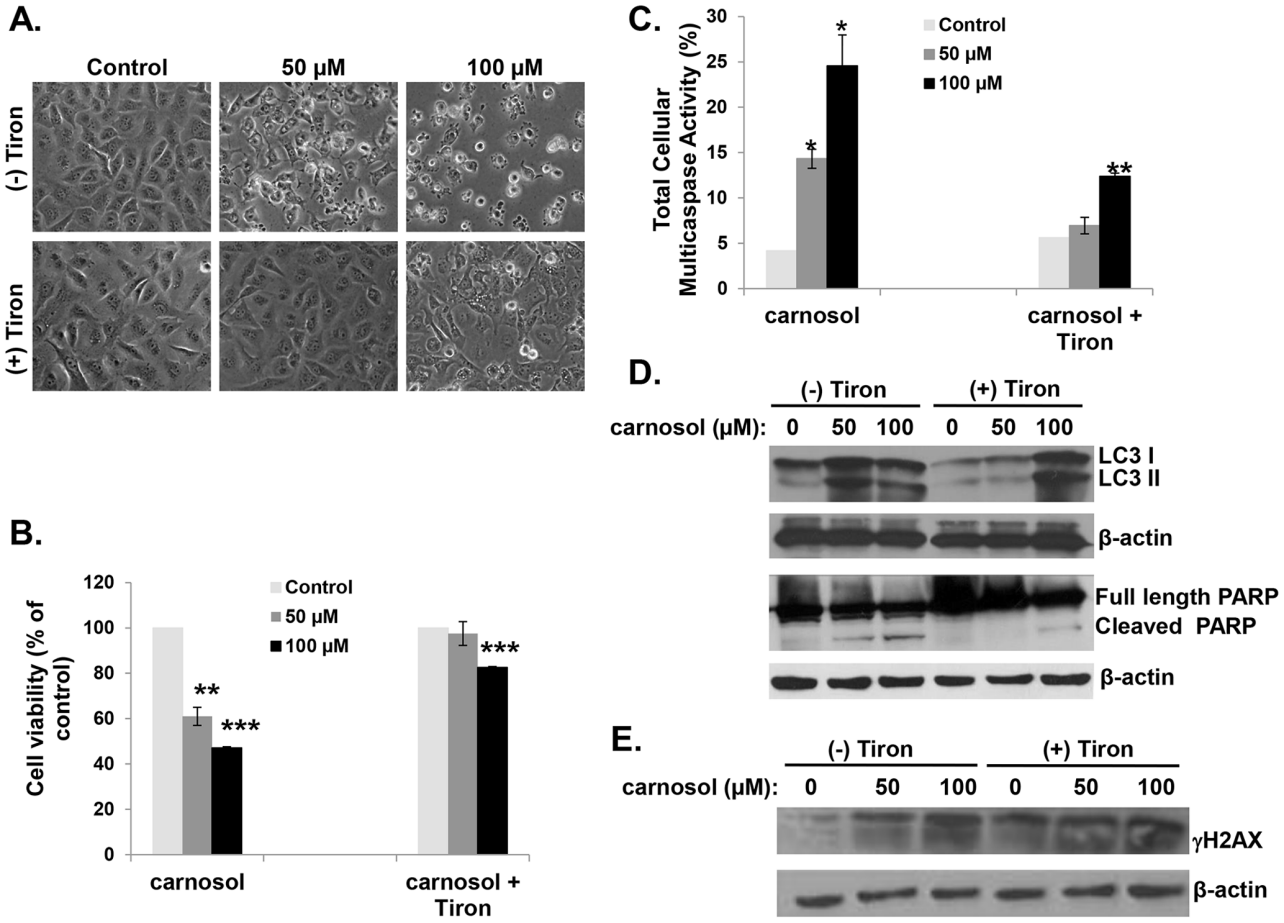
The ROS scavenger tiron attenuates carnosol-mediated autophagy, apoptosis and DNA damage in MDA-MB-231 cells. (**A**) Micrograph observation of MDA-MB-231 cells pretreated with or without tiron for 1 h and then treated with 50 and 100 µM carnosol for 24 h. (**B**) Cells pretreated with or without tiron for 1 h and then treated with 50 and 100 µM carnosol for 24 h. Cell viability was determined with the Muse Cell Analyzer as described by the manufacturer. Cell viability assay was performed in triplicate and results obtained represent the average of three independent experiments. (**C**) Multicaspase activity in MDA-MB-231 cells pretreated with carnosol for 1 h and then treated with 50 and 100 µM of carnosol for 24 h. Multicaspase activity was analyzed using the Muse Multicaspase assay kit and quantified on the Muse Cell Analyzer. Total cellular caspase activity assay was performed in triplicate and results obtained represent the average of three independent experiments. (**D**) Western blotting analysis of LC3II accumulation, PARP cleavage and (**E**) γH2AX expression in MDA-MB-231 cells pretreated with or without tiron for 1 h and then treated with 50 and 100 µM of carnosol for 24 h.

Although ROS is known to induce autophagy and apoptosis, evidence that autophagy also triggers ROS exists [28]. Our data showed that the accumulation of ROS become evident as early as 1 h post-treatment with carnosol (figure 6B), while autophagy, examined through LC3II accumulation, started after 3 h (Figure 5D) suggesting that ROS generation is upstream of autophagy induction. In order to examine the role of ROS generation in autophagy and apoptosis induction, we examined the effect of tiron, a ROS scavenger, on cell viability, caspases activation, PARP cleavage and LC3II accumulation. Light microscopy observation revealed that cells pre-treated with tiron for 1 h prior to treatment with 50 µM carnosol were indistinguishable from the control cells as no sign of vacuolation, nor apoptotic cells were observed (figure 7A). Similarly the effect of 100 µM carnosol was also attenuated by tiron as much less apoptotic cells could be observed and only few cells showed vacuolation (figure 7A). Next, MDA-MB-231 cells were treated with 50 or 100 µM carnosol with and without tiron and cell viability was determined as described in Material and Methods. As it is shown in figure 7B, inhibition of ROS generation completely reversed the effect of 50 µM carnosol on cell viability. Our data showed that a concentration of 50 µM of carnosol has no effect on cell viability when cells were pre-treated with tiron. Similarly, tiron dramatically reduced the effect of 100 µM carnosol. Cell survival shifted from 50% with carnosol alone, to 80% when tiron was present. To assess for apoptosis, we first treated MDA-MB-231 cells with 50 and 100 µM with and without tiron and measured total caspases activity in the cells. Figure 7C, showed that, in absence of tiron, 50 and 100 µM carnosol led to a concentration-dependent activation of total cellular multicaspases. However, when tiron was present, 50 µM carnosol showed no changes in multicaspase activity pattern compared with control. Absence of caspases activation was also confirmed by the absence of PARP cleavage (figure 7C) indicating absence of cell death in these cells (Figure 7D). Similarly, Tiron also reduced the effect of 100 µM carnosol on apoptosis induction revealed by less cellular multicaspase activation (Figure 7C) and reduced PARP cleavage (Figure 7D) compared to cells treated with carnosol alone. Taken together, these data strongly argue in favor of ROS-dependent induction of apoptosis in MDA-MB-231 cells.

A link between ROS production and autophagy induction was also investigated. We found that attenuation of ROS level by tiron significantly decreased the level of LC3II, a hallmark for autophagy induction. Western blot analysis revealed that the level of LC3II accumulation observed in cells treated with 50 µM in presence of tiron was comparable to that of control (Figure 7D), indicating the absence of autophagy in these cells, in agreement with light microscopy observation which showed no vacuolation. On the other hand, a concentration of 100 µM, did not suppress but only attenuated autophagy, again in agreement with light microscopy observation which revealed vacuolation. Reduction of apoptosis but not of autophagy by tiron at a concentration of 100 µM carnosol could be explained by the fact that the quantity of ROS induced by this concentration of carnosol exceeds the scavenging capacity of tiron. As result, we believe that tiron reduces the intracellular concentration of ROS to a level enough to trigger autophagy but below a threshold required for activation of massive apoptosis. These data strongly suggest that the intracellular concentration of ROS determines the response of breast cancer cells toward either autophagy only or autophagy and apoptosis.

Excess ROS production was shown to cause various cellular damages including DNA damage. Conversely, DNA damage resulting from different sources was also shown to increase ROS levels, which in turn contribute to the induction of cell death. Because we found that increasing intracellular ROS generation correlated with the activation of the marker of DNA damage, γH2AX (Figure 6C and 6D), we wanted to determine which of the two events, ROS production or DNA damage, occurred first in carnosol-treated breast cancer cells. As it is shown in figure 7E, inhibition of ROS production by tiron, attenuated the activation of γH2AX and hence DNA damage in MDA-MB-231 cells, indicating that DNA damage occurred downstream of ROS production. Collectively, these results demonstrate that carnosol exerts its anti-breast cancer effect through a ROS-dependent mechanism(s) that includes DNA damage, and that inhibition of ROS generation is enough to block both carnosol-induced autophagy and apoptosis.

### Carnosol Inhibits Colony Growth of MDA-MB-231

**Figure 8:**
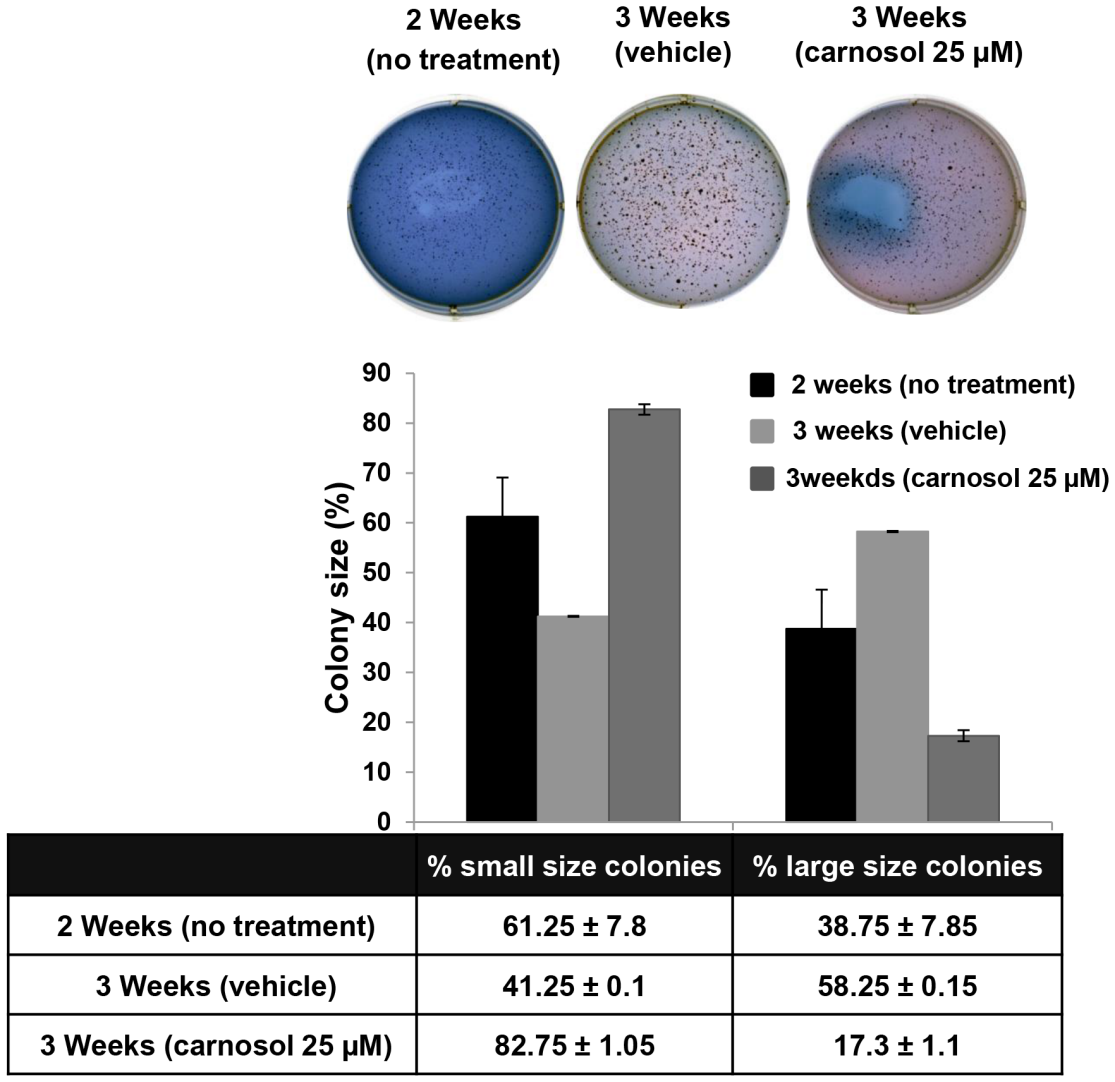
Carnosol inhibits MDA-MB-231 colony growth. Carnosol induced arrest of MDA-MB-231 colony growth. MDA-MB-231 colonies were first allowed to form in normal media for 2 weeks as described in Material and Methods. Formed colonies were then treated with DMSO or 25 µM carnosol and allowed to grow for one more week before staining. Inhibition of colony growth was assessed by measuring the size of the colonies obtained in DMSO and carnosol-treated plate. Data were compared with those obtained for the 2 weeks colonies. The size of the colonies were measured, counted using a microscope (10×) and the colony size was categorized as Large (≥200 µM), or small (50–200 µM). Student's t test was performed to determine the significance (*p<0.05, **p<0.01 and ***p<0.005).

To further confirm the inhibitory potential of carnosol on MDA-MB 231 cells, we sought to determine if carnosol could inhibit the further growth of already formed MDA-MB-231 colonies. For this purpose, MDA-MB-231 cells were first allowed to grow and form visible colonies in absence of treatment. After 14 days of growth, colonies were incubated with DMSO as control or 25 µM carnosol and allowed to grow for one more week. Figures 8 shows that the size of the DMSO-treated (control) colonies kept growing compared to the size of the two weeks colonies; more large colonies were obtained in the three weeks plate, while less small colonies were counted, indicating that small colonies became larger in size. On the other hand, carnosol-treated colonies shows regression in colony size compared to the two weeks colonies. In fact, in the carnosol-treated plates, the number of large size colonies counted at three weeks (17.3%) was less than what was obtained in the two weeks plate (38.75%), while the number of small colonies was significantly greater going from 61.25% at two weeks to 82.75% at three weeks, suggesting size regression in the large colony in response to carnosol treatment. This result along with the viability and cell cycle data confirm the anti-cancer effect of carnosol on the MDA-MB-231 breast cancer cells.

## Discussion

The triple negative breast cancer (TNBC) is considered as highly aggressive form of cancer with poor survival rate for the patients [29]. Currently, treatment of TNBC is mainly through conventional chemotherapy which showed limited long-term success [30]. Hence, Identification of new more effective therapeutic compounds against TNBC remains an important clinical challenge.

In the present study we investigated the effectiveness of carnosol, a natural compound, against the TNBC MDA-MB-231 cells. We showed that carnosol efficiently inhibited *in vitro* and *in vivo* the growth of the MDA-MB-231 cells. We found that carnosol induced cell cycle arrest at G2 phase confirmed by a decrease in p(ser10) histone H3 . G2 arrest by carnosol was also shown in prostate cancer PC3 cells [17] and was associated with an upregulation of the CDK inhibitors p21 and p27. In MDA-MB-231 cells, however, we found that the cell cycle arrest correlated with an upregulation of the CDK inhibitor p21 and down regulation of p27, thus suggesting that carnosol-induced cell cycle arrest in TNBC might involves different mechanism(s) and does not require p27. Similar to carnosol, salinomycin, a monocarboxylic polyether antibiotic, was also shown to induce G2 arrest, upregulation of P21 and downregulation of p27 in MDA-MB-231 cells [31]. We also found that arrested MDA-MB-231 cells, in response to carnosol, undergoes apoptosis. This finding is in agreement with previous reports demonstrating that the anticancer effect of carnosol on leukemia [19], prostate cancer [15], [17] and colon cancer [18] cell lines was due to its apoptosis-inducing activity. Interestingly, our results showed for the first time that carnosol activated both intrinsic and extrinsic apoptotic pathway in TNBC through an activation of caspase 9 and 8 respectively. Carnosol induced expression of pro-apoptotic protein with concomitant decrease of Bcl2 expression and loss of mitochondrial membrane potential.

Increasing number of anticancer therapies has been shown to induce autophagy in different cancer cell types [32]. Still, whether autophagy in response to anticancer therapies is pro-death or pro-survival remains subject to debate. However, there are increasing evidences that Beclin1-independent autophagy is invariably associated with cell death [33], [34]. In the present study, we found that carnosol also induced autophagy in MDA-MB-231. This finding is supported by large body of evidence, (i) Electron micrograph observation of autophagy features such as massive mitophagy and cytoplasmic vacuolation, (ii) modulation of autophagy-specific markers such as conversion of LC3 I to LC3 II and modulation of p62(SQSTM1) accumulation. To our knowledge this is the first study to show that carnosol induces autophagy in cancer cell lines. We found that autophagy induction, which occurred as early as 3 h post-treatment, precedes the activation of the programmed cell death which took place at 24 h post-treatment. Moreover, electron micrograph analysis revealed the existence of both events, i.e. autophagy and apoptosis, within the same cell. Simultaneous induction of autophagy and caspase-dependent apoptosis in cancer cells was also described for several anticancer compounds such canabidiol in the breast cancer MDA-MB-231 cells [36], microtubule-modulating agent, Red-Br-nos [35] and oridonin [36] in PC3 prostate cancer cells. To our knowledge, this is the first report that shows that carnosol induces autophagy in cancer cells. Strikingly we found that carnosol-induced autophagy is Beclin1-independent. In fact the level of Beclin1 remained unchanged under all experimental conditions used of concentrations or time of exposure (data not shown), hence demonstrating that carnosol-mediated autophagy is beclin1-independent in MDA-MB-231 cells. Although Beclin1 was most of the time perceived as key player in autophagy execution and its knock down blocks autophagic cell death [37], a recent study by Wong and collaborators showed that small molecule compound, referred as C1, that triggers intracellular ROS production, induced simultaneous induction of Beclin1-independent autophagy and apoptosis in various cancer cell type including MDA-MB-231 breast cancer cells [21]. These authors also showed that sustained ERK1/2 activation act as an upstream effector controlling both autophagy and apoptosis in response to high level of intracellular ROS, and that its pharmacological inhibition blocked the C1 induced autophagy and apoptosis [21]. Interestingly, here we showed that carnosol induced both ROS production and sustained ERK1/2 activation hence strongly suggesting that carnosol might exerts its cytotoxic effect on TNBC at least partly through ERK1/2 activation. This study adds carnosol to the increasing list of anticancer compounds that induce Beclin1 independent autophagy in tumor cells.

There is increasing number of evidences highlighting the central role of ROS production in inducing autophagy and cell death in many cancer cell types. In fact, several anticancer agents were shown to mediate their effect through ROS, and inhibition of ROS production by ROS scavenger blocks autophagy and cell death in many cancer types [34, 35, 38, and 39]. Similarly, in this study we observed carnosol-enhanced ROS generation in breast cancer cells in dose- and time-dependent manner. Recent studies showed that the extent of ROS level produced, in response to anticancer agents, elicits different responses in cancer cells. While low level of ROS was shown to induce autophagy, excessive ROS accumulation triggered both apoptosis and cell death. In agreement with these studies, we found that, depending on the concentration and time of exposure, carnosol elicited different responses in MDA-MB-231 cells. We found that carnosol at non cytotoxic concentration (25 µM) resulted in low level in ROS production and γH2AX activation, and induced autophagy. We believe that in this context, autophagy was induced to remove damaged organelles from treated cells. However, exposure of cells, for short period of time, to higher concentration of carnosol (≥50 µM) triggered autophagy as self-defense survival mechanism. A prolonged exposure to such concentration of carnosol led to excessive ROS production, which ultimately resulted in higher levels of oxidative damage that exceeds the cell's repair capabilities that eventually caused programmed cell death through activation of intrinsic and extrinsic apoptotic pathways. These findings which highlight the essential role of ROS accumulation are supported by the following evidence: The abrogation of ROS production by the ROS scavenger tiron, totally blocked autophagy and apoptosis in cells treated with 50 µM carnosol and significantly attenuated both events at a higher concentration (100 µM). Altogether, these data strongly demonstrate that ROS production in response to carnosol act as an upstream effector for autophagy and subsequent apoptosis induction. Similar mechanism has been described by Lin and collaborators for safingol, an anticancer drug in phase I clinical trial, which has been shown to mediate a concentration-dependent effect in MDA-MB-231 and HT29 cancer cells [38]. These authors showed that Low concentration of safingol triggered autophagy as damage repair mechanism, while higher concentration led to cell death [38].

DNA damage caused by genotoxic chemicals or ROS accumulation was shown to induce autophagy. Still the mechanisms by which DNA damage triggers autophagy are unclear [40]. Interestingly, we found that carnosol induced a dose-dependent increase in γH2AX, a marker of DNA damage, detected as early as 3 h, a time that coincides with autophagy induction. Most importantly, we found that inhibition of ROS production by tiron attenuated the activation of γH2AX. Bases on these finding, we demonstrate that DNA damage is a downstream response to ROS production that might contribute to induce autophagy and apoptosis in MDA-MB-231 cells.

Nowadays, much attention is directed toward the potential role of mitochondria damage in autophagy induction. The current hypothesis is that cells respond to mitochondrial damage in a graded fashion: when only a few mitochondria are damaged, autophagy takes place and the mitochondria are degraded; when more mitochondria are damaged, apoptosis is induced, and the cells die [32]. In agreement with this hypothesis, our electron micrograph data clearly revealed that carnosol affected the mitochondrial morphology in dose-dependent manner which ultimately resulted in loss of mitochondrial membrane potential. Thus, the induction of autophagy with or without subsequent apoptosis activation might reflect the extent of mitochondria damage in carnosol-treated cells.

**Figure 9:**
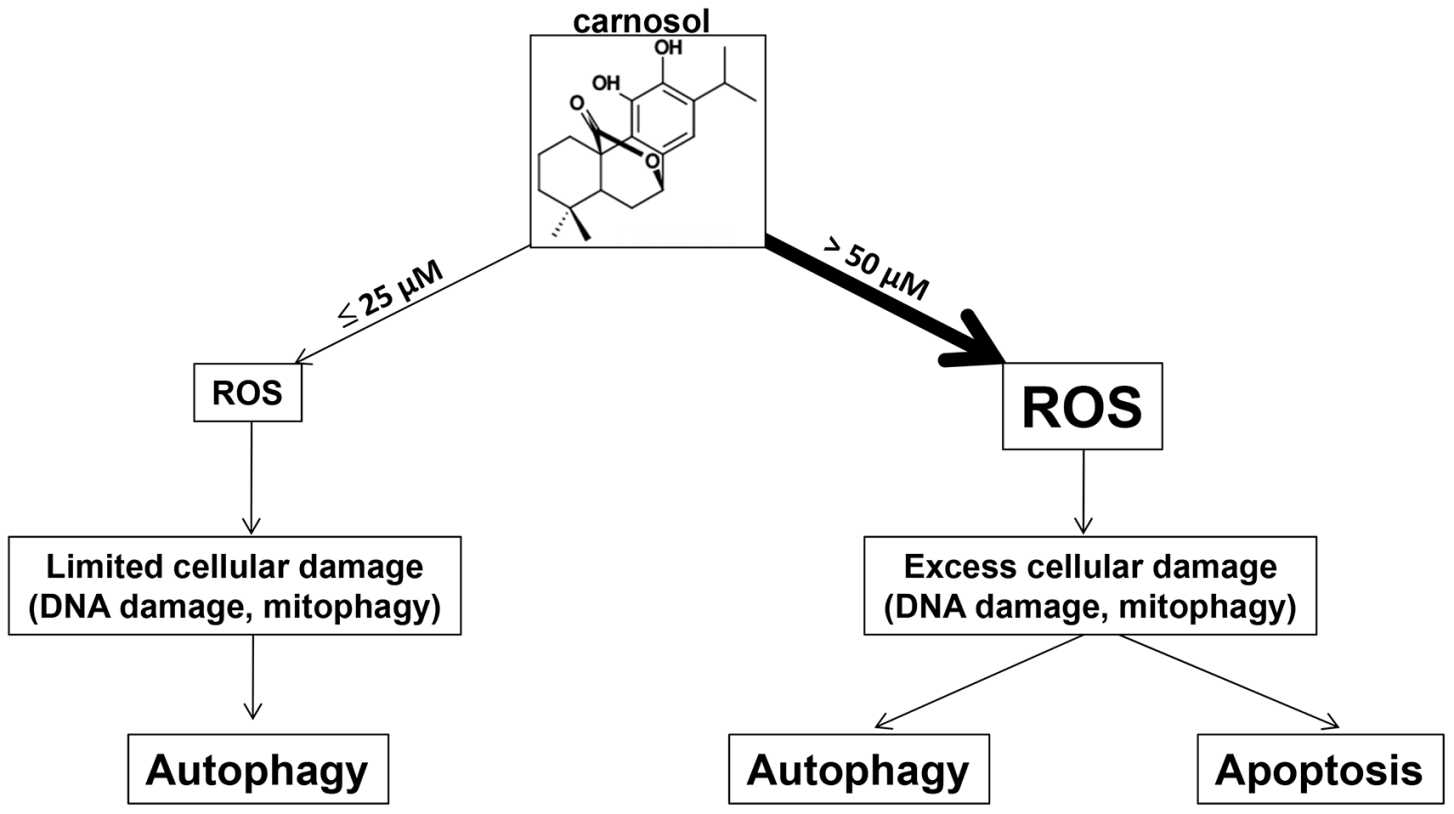
Hypothetic model illustrating the differential effect of carnosol in MDA-MB-231 breast cancer cells.

In conclusion, our data are consistent with a model shown in figure 9, in which treatment with carnosol triggers oxidative damage to TNBC. The magnitude of damage, which depends upon the concentration of carnosol, determines the response of the cells. We propose that in the presence of large amounts of intracellular damages induced by high concentration of carnosol, MDA-MB-231 cells respond by triggering autophagy with subsequent apoptosis through an activation of both intrinsic and extrinsic pathway. In contrast, limited intracellular damage caused by low concentration of carnosol triggers only autophagy as repair mechanism. Our current study provides experimental evidence that the plant-derived carnosol can be promising candidate for triple negative breast cancer therapy.

In the present study, we investigated the effect of carnosol on the highly proliferative and invasive triple-negative MDA-MB-231 human breast cancer cells. We show for that carnosol blocked cell cycle at G2 phase and induced ROS-dependent apoptosis and beciln1-independent autophagy in MDA-MB-231 breast cancer cells.
