# Correction: Carnosol Induces ROS-Mediated Beclin1-Independent Autophagy and Apoptosis in Triple Negative Breast Cancer

**DOI:** 10.1371/journal.pone.0337572

**Published:** 2025-11-26

**Authors:** Yusra Al Dhaheri, Samir Attoub, Gaber Ramadan, Kholoud Arafat, Khuloud Bajbouj, Noushad Karuvantevida, Synan AbuQamar, Ali Eid, Rabah Iratni

There are errors in Figs 3 and 8. Specifically,

- In Fig 3A, the Early Apop. values reported in the lower right quadrants of the Control, 50 µM, and 100 µM panels are incorrect. A corrected Fig 3 is provided with this notice and underlying the flow cytometry images are provided in S1 File.
- In Fig 8:
  - There are errors in the values reported in the table for the SEM of % small size colonies in the 2 weeks (no treatment) group and for the mean % small size colonies in the 3 weeks (vehicle) group.
  - The figure caption incorrectly states that “Student’s t test was performed to determine the significance (*p<0.05, **p<0.01 and ***p<0.005)”; however, no statistical analyses were performed for the colony formation assay in Fig 8.
  - The well image for 2 weeks (no treatment) is a duplicate of an image previously published in Fig 8 of [2]; the well image for 3 weeks (vehicle) is a duplicate of an image previously published in Fig 4B of [3]. The authors clarify that these images correctly represent the reported conditions; three studies of different compounds were run in parallel and share the same control wells.
  - An updated Fig 8, including the corrected values and legend, along with original underlying quantitative data (S2 File) are provided with this notice. The updated Fig 8 presents alternative replicate control well images from the original study; the underlying colony formation assay images for 3 Weeks (Carnosol) are provided in S1 File.

The following additional issues were reviewed by the *PLOS One* Editors and are considered resolved.

There are similarities in appearance between:

- The β-Actin panel in Fig 2C of [1] and the mTOR panel in Fig 3C of a later publication [4]
- The β-Actin for cyclin in Fig 2D of [1] and the AMPK panel in Fig 3C of a later publication [4]

The *PLOS One* Editors reviewed the underlying blots for Fig 2 of [1] (provided here in S3 File) which support the provenance of the data in Fig 2.

The authors confirm that the underlying data for all parts of the study are available upon request.

The originally published Fig 8 panel 3 Weeks (Vehicle), reports material from [3], published in 2013 by Elsevier, which are not offered under a CC BY license and are therefore excluded from this article’s [1] license.

**Fig 3:**
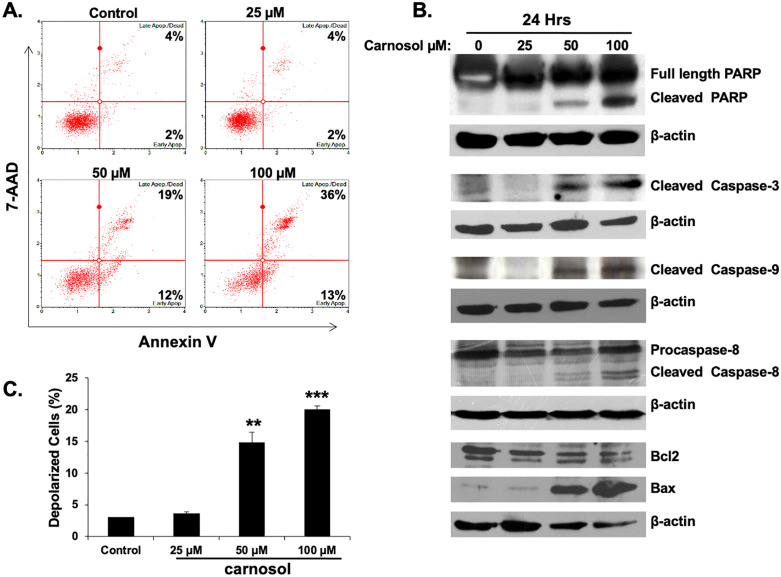
Induction of caspases-mediated apoptosis by carnosol in the MDA-MB-231 cells. **(A)** Carnosol induced apoptosis in the MDAMB-231 cells. Annexin V binding was carried out using Annexin V & Dead Cell kit (Millipore). Cells were treated with DMSO or various concentration of carnosol for 24 **h.** Detached and adherent cells were collected and stained with Annexin V and 7-AAD and then the events for early and late apoptotic cells were counted with the Muse Cell Analyzer as described in Materials and Methods. **(B)** Western blot analysis of caspase 3, −9, and −8 activation, PARP cleavage and Bcl1 and Bax expression in MDA-MB-231 cells were treated with increasing concentrations of carnosol (25, 50 and 100 mM) for 24 **h. (C)** Carnosol induces the depolarization of mitochondrial membrane. Mitochondrial membrane potential (MMP) was assessed with the Muse Cell Analyzer using the Muse MitoPotential kit as described in Materials and Methods. Data represent the mean SEM of at least 3 independent experiments. Student’s t test was performed to determine the significance (*p <  0.05, **p < 0.01 and ***p < 0.005).

**Fig 8:**
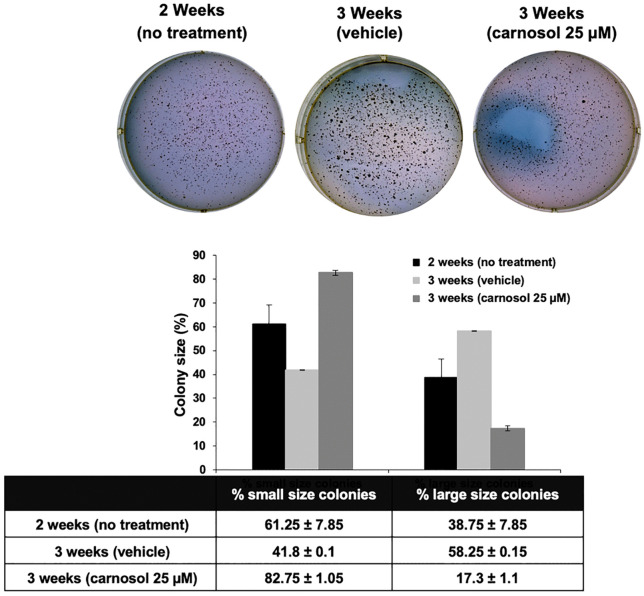
Carnosol inhibits MDA-MB-231 colony growth. Carnosol induced arrest of MDA-MB-231 colony growth. MDA-MB-231 colonies were first allowed to form in normal media for 2 weeks as described in Material and Methods. Formed colonies were then treated with DMSO or 25 µM carnosol and allowed to grow for one more week before staining. Inhibition of colony growth was assessed by measuring the size of the colonies obtained in DMSO and carnosol-treated plate. Data were compared with those obtained for the 2 weeks colonies. The size of the colonies was measured, counted using a microscope (10X) and the colony size was categorized as Large (>200 µm), or small (50-200 µm).

## Supporting information

S1 File Original, uncropped images of the 3 Weeks (Carnosol) wells used in the colony formation assays, as referenced in Figure 8. Screenshots of the flow cytometer output, used to create Fig 3A.
(ZIP)

S2 File Original data underlying the colony size table and chart referenced in Fig 8. (XLSX)

S3 File Original, uncropped images of the western blots referenced in Fig 2. (PDF)
