# Supplementary material for: Correction: Carnosol Induces ROS-Mediated Beclin1-Independent Autophagy and Apoptosis in Triple Negative Breast Cancer
Source: PLoS One. 2025 Nov 26;20(11):e0337572. doi: 10.1371/journal.pone.0337572 (PMC12654894; doi:10.1371/journal.pone.0337572)
Supplement: S1 File — Screenshots of the flow cytometer output, used to create Fig 3A. (ZIP) [file pone.0337572.s001.zip › 3 weeks carnosol-treatment wells.pdf]

Well for 3 weeks (carnosol) Replicate 1

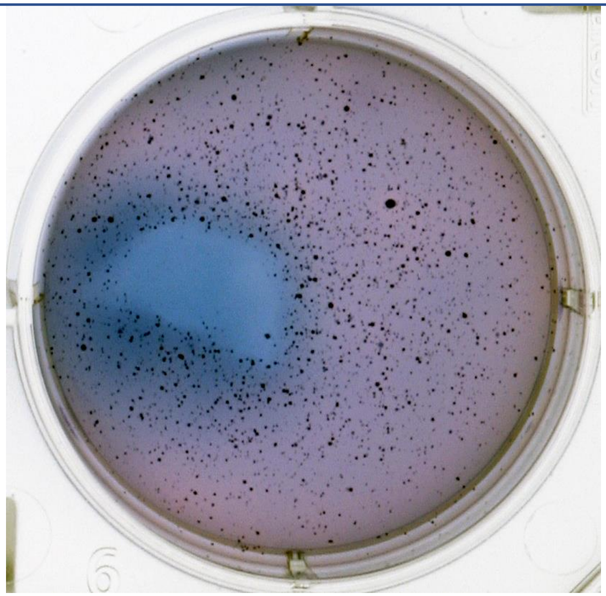

Picture 3 weeks carnosol treatment used for Carnosol paper

Well for 3 weeks (carnosol) Replicate 2

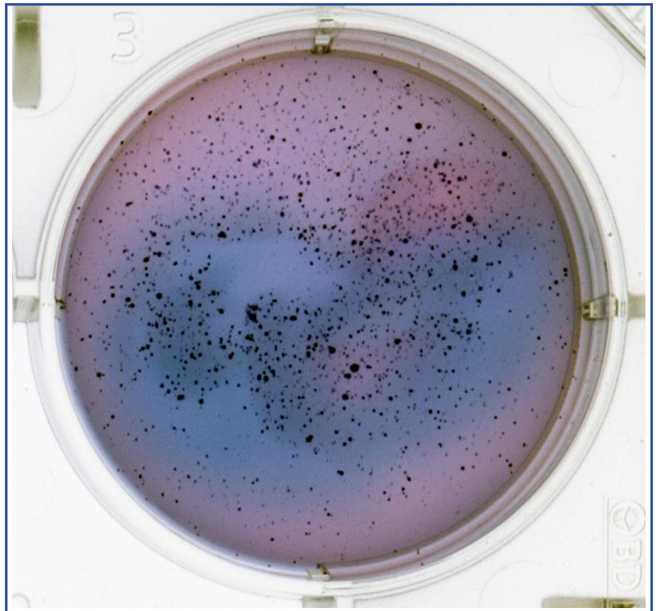

Picture 3 weeks carnosol treatment.
