# Supplementary material for: Correction: Carnosol Induces ROS-Mediated Beclin1-Independent Autophagy and Apoptosis in Triple Negative Breast Cancer
Source: PLoS One. 2025 Nov 26;20(11):e0337572. doi: 10.1371/journal.pone.0337572 (PMC12654894; doi:10.1371/journal.pone.0337572)

Original blot for figure 2C (H3pSer10 and its corresponding beta actin)

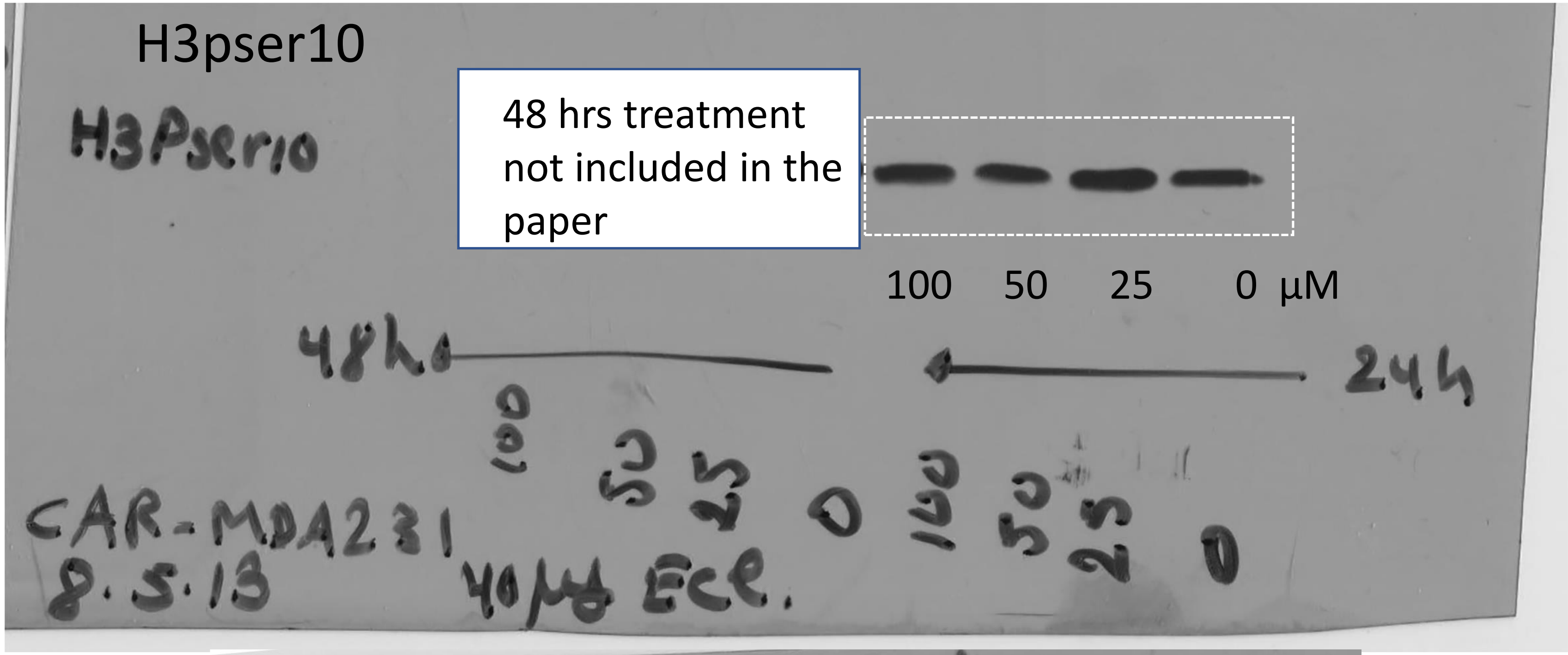

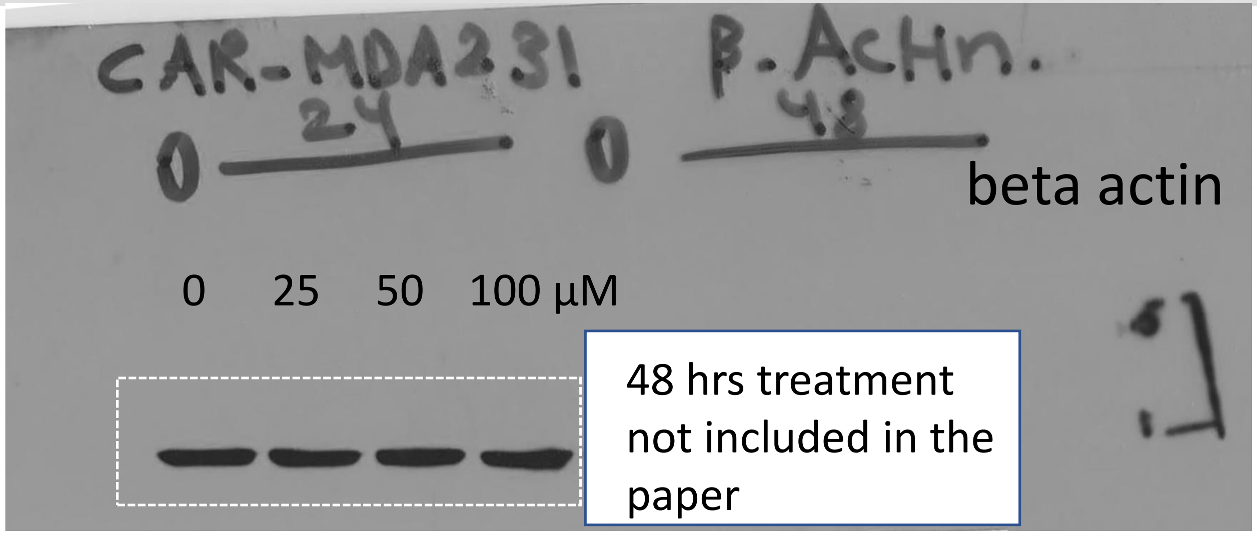

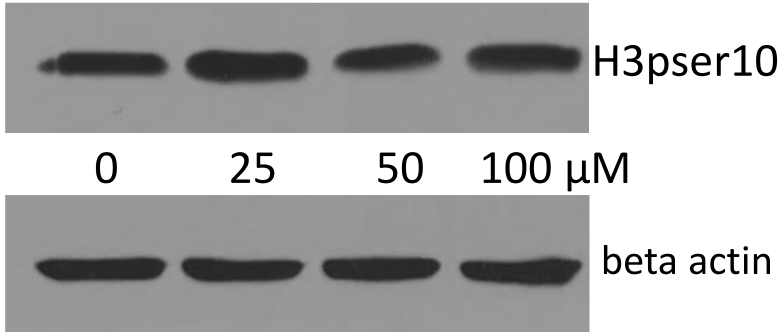

Original blot for figure 2D (Cyclin B1and its corresponding beta actin)

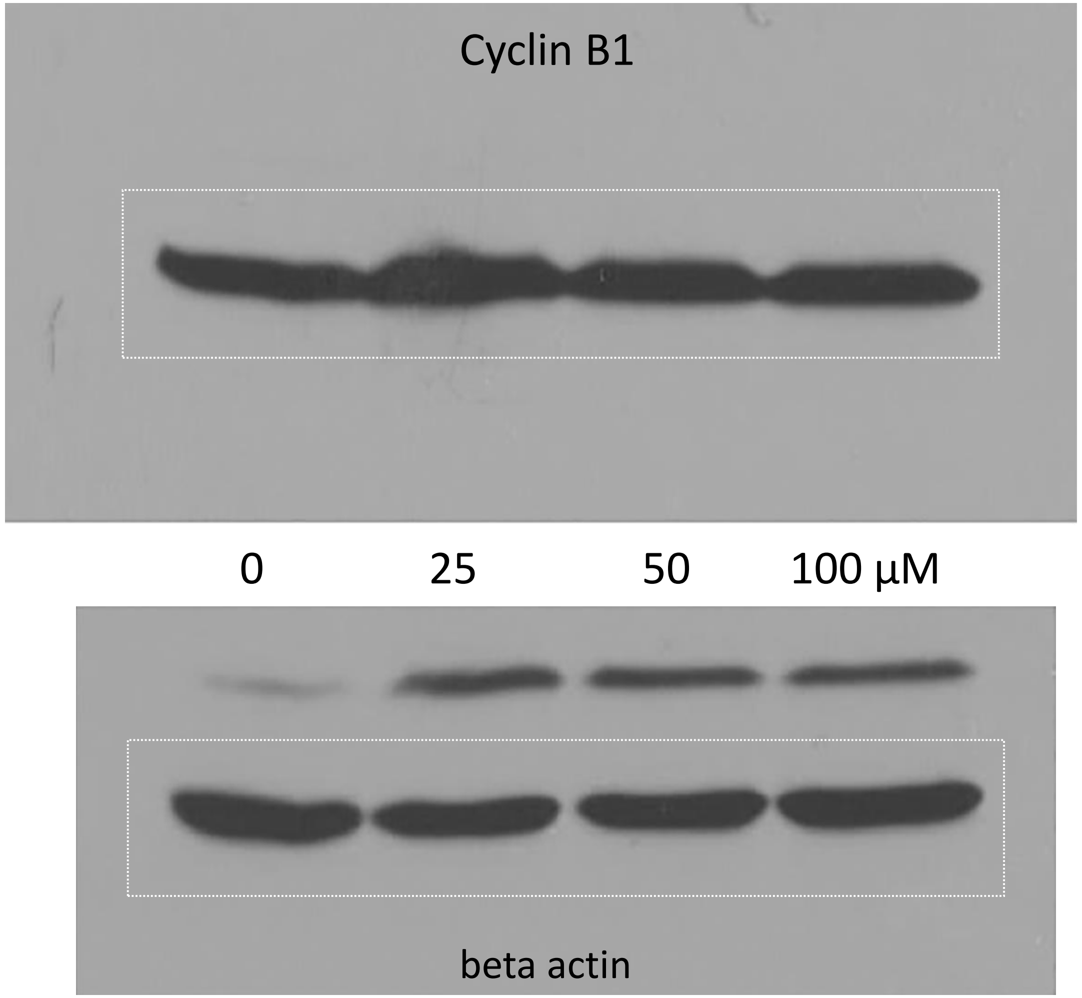

Original blot for figure 2D (p21 and p27 and corresponding beta actin)

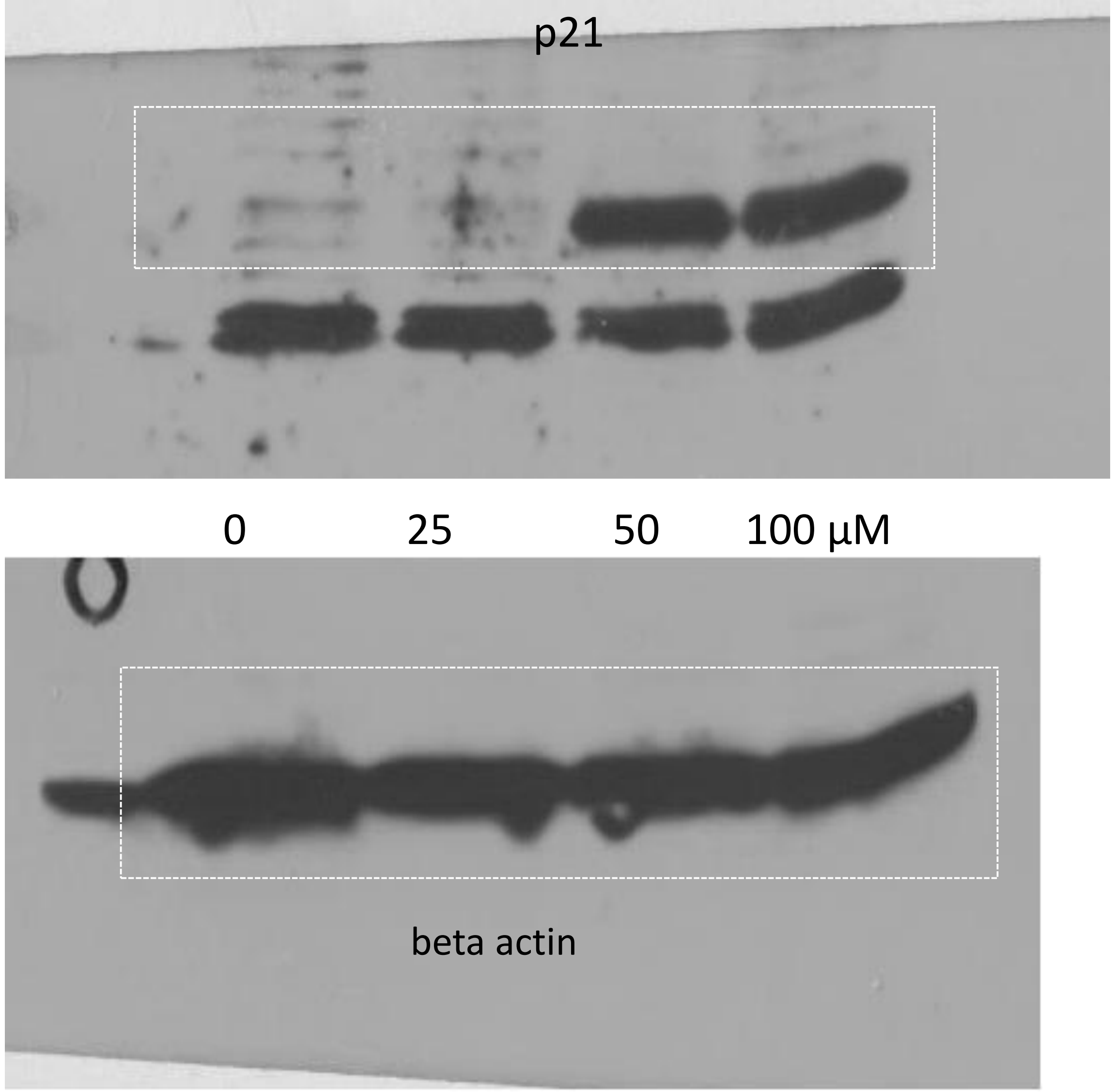

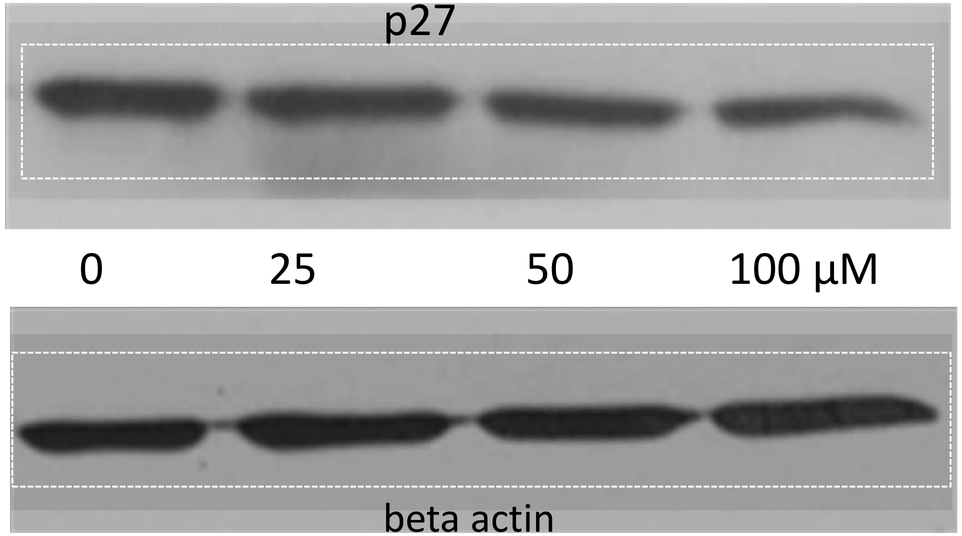

Supplement: S3 File — (PDF) [file pone.0337572.s003.pdf]
